# Novel tri-specific tribodies induce strong T cell activation and anti-tumor effects in vitro and in vivo

**DOI:** 10.1186/s13046-022-02474-3

**Published:** 2022-09-07

**Authors:** Margherita Passariello, Asami Yoshioka, Kota Takahashi, Shu-ichi Hashimoto, Toshikazu Inoue, Koji Nakamura, Claudia De Lorenzo

**Affiliations:** 1 Department of Molecular Medicine and Medical Biotechnologies, University of Naples “Federico II”, 80131 Naples, Italy; 2 Ceinge - Biotecnologie Avanzate S.C. a.R.L, via Gaetano Salvatore 486, 80145 Naples, Italy; 3 Chiome Bioscience Inc, 3-12-1 Honmachi Shibuya-Ku, Tokyo, 151-0071 Japan

**Keywords:** Immunotherapy, Bi-Specific T Cell Engager, Tri-Specific Tribodies, Immune Checkpoints, Cancer

## Abstract

**Background:**

Immunotherapy based on Bi-specific T Cell Engagers (TCE) represents one of the most attractive strategy to treat cancers resistant to conventional therapies. TCE are antibody-like proteins that simultaneously bind with one arm to a Tumor Associated Antigen (TAA) on cancer cells and with the other one to CD3 complex on a T-cell to form a TCR-independent immune synapse and circumvent Human Leucocyte Antigen restriction. Among them, the tribodies, such as Tb535H, a bi-specific molecule, made up of a Fab and a scFv domain both targeting 5T4 and another scFv targeting CD3, have demonstrated anti-tumor efficacy in preclinical studies.

**Methods:**

Here, we generated five novel tri-specific and multi-functional tribodies, called 53X tribodies, composed of a 5T4 binding Fab arm and a CD3 binding scFv, but differently from the parental Tb535H, they contain an additional scFv derived from an antibody specific for an immune checkpoint, such as PD-1, PD-L1 or LAG-3.

**Results:**

Compared with the parental Tb535H bi-specific T cell engager targeting 5T4, the novel 53X tribodies retained similar binding properties of Tb535H tribody, but showed enhanced anti-tumor potency due to the incorporation of the checkpoint inhibitory moiety. In particular, one of them, called 53L10, a tri-specific T cell engager targeting 5T4, CD3 and PD-L1, showed the most promising anti-tumor efficacy in vitro and led to complete tumor regression in vivo.

**Conclusions:**

The novel tribodies have the potential to become strong and safe therapeutic drugs, allowing to reduce also the cost of production as one single molecule contains three different specificities including the anti-TAA, anti-CD3 and anti-IC binding arms.

**Supplementary Information:**

The online version contains supplementary material available at 10.1186/s13046-022-02474-3.

## Background

Bi-specific, CD3-based T Cell Engagers (TCE) are antibodies or antibody-like proteins that simultaneously bind with one arm to a Tumor Associated Antigen (TAA) on cancer cells and with the other one to CD3 complex on a T-cell to form a TCR-independent artificial immune synapse and circumvent Human Leucocyte Antigen (HLA) restriction. TCE represents one of the most attractive modalities to treat cancers resistant to conventional therapies [1–5]. In the field of hematological malignancies, Blinatumomab is a Bi-specific T Cell Engager (BiTE) that simultaneously targets CD19 antigen on the Acute Lymphoblastic Leukemia (ALL) and CD3 on T- cells [6, 7] to induce efficient killing of leukemia cells (FDA approval in 2014). On the other hand, significant challenges in treating solid tumors with TCEs are represented by the immunosuppressive Tumor Microenvironment (TME). Solid tumors recruit immunosuppressive cells such as Myeloid Derived Suppressor Cells (MDSCs), Tumor-Associated Macrophages (TAMs), and regulatory T cells (Tregs) to form TME, all of which inhibit the activity of cytotoxic T cells [8, 9].

Therefore, the most effective use of TCEs in solid tumors will likely require the use of TCEs combined with agents such as Immune Checkpoint Inhibitors (ICIs) that help to overcome the immunosuppressive TME and render an immune excluded or immune desert “cold” tumor into an inflamed “hot” one [10]. Ideally a single molecule could be designed that is able to have both the functions of TCE and of ICIs, so that this novel construct will allow to overcome the immunosuppressive TME in solid tumors and to lower the cost of production with respect to that of two separate drugs to be combined.

The oncofetal tumor-associated antigen 5T4, also known as TPBG or WAIF1, is a 72 kDa cell surface glycoprotein containing eight leucine-rich repeats [11]. 5T4 is expressed in fetal trophoblasts as early as 9 weeks of gestation. The expression of 5T4 in normal adult is restricted to only a few specialized epithelial cell types, such asepithelium of bladder, villous epithelium of small intestine, endometrial gland of uterus, endocervical glands of cervices and basal epidermis of skin even though, the level of expression of these tissues was found to be at least 1,000-fold lower than that observed in the placenta [12, 13].

On the other hand, it has been reported that 5T4 is expressed in a wide variety of cancer types including ovarian, colon, cervical, breast, gastric and lung cancers [14–18]. In addition, its expression has been observed also on bladder, endometrium, oesophagus, pancreas, stomach and testicular non-seminomatous germ cell tumors. Therefore, 5T4 has been suggested as a suitable target antigen for targeted cancer therapy [19].

Bi-specific antibodies that engage 5T4 on tumor cells and CD3 expressed on T cells may represent an interesting strategy to redirect cytotoxic T cells against cancer cells for inducing efficient killing. There are some bi-specific antibodies targeting 5T4 on the tumor cells and CD3 or 4-1BB, which is a co-stimulatory molecule on T cells, to treat solid tumors in clinical trials [20]. However, to date, no anti-5T4 immunotherapy has been approved for treating cancer.

The tribody format is a multi-specific antibody including a Fab domain as a scaffold, in which Fd fragment of H-chain (V_H_ + CH_1_) and L-chain (V_L_ + C_L_) can naturally heterodimerize in vivo, and additional functions such as single-chain variable fragments (scFvs) can be incorporated onto the scaffold [21, 22]. Among the tribodies, Tb535H is a bi-specific tribody, made up of a Fab and a scFv domain both targeting 5T4 (bivalent binding) and another scFv targeting CD3 (monovalent binding) [23]. Tb535H is now under clinical testing in the phase I study for solid tumor treatment (jRCT2031210708).

Here, we generated five novel tri-specific and multi-functional tribodies, called 53X tribodies, composed of 5T4 binding Fab arm and CD3 binding scFv, but differently from the parental Tb535H, they contain an additional scFv derived from an antibody specific for an immune checkpoint molecule, such as PD-1, PD-L1 or LAG-3 [24–28]. Compared with parental Tb535H, which is a bi-specific T cell engager targeting 5T4, the novel 53X tribodies not only retained similar binding properties of the parental Tb535H tribody, but showed enhanced anti-tumor potency by incorporating also checkpoint inhibition in a single molecule in addition to 5T4-mediated TCR/CD3 activation. In particular, one of the novel tribodies, called 53L10, a tri-specific T cell engager targeting 5T4, CD3 and PD-L1 IC, showed the most promising anti-tumor efficacy in vitro and in vivo.

## Methods

### Cell cultures

MDA-MB-231, BT-549 and MCF-7 breast cancer cells were cultured in Dulbecco’s Modified Eagle’s Medium (DMEM Gibco, Life Technologies, Paisley, Scotland UK), Roswell Park Memorial Institute 1640 Medium (RPMI 1640, Gibco, Life Technologies, Paisley, UK) and Modified Eagle’s Medium (MEM, Gibco, Life Technologies, Grand Island, USA), respectively. A-549 lung cancer cells were cultured in Kaign’s Modification of Ham’s F-12 Medium (F-12 K Gibco, Life Technologies, Paisley, Scotland UK). The media were supplemented with 10% (vol/vol) heat-inactivated fetal bovine serum (FBS Sigma-Aldrich, St Louis, Missouri USA) and were used after addition of 50 U/mL penicillin, 50 μg/mL streptomycin, 2 mM L-glutamine (all from Gibco Life Technologies, Paisley, Scotland UK). HuT 78 cutaneous T lymphocyte cell line was cultured in Iscove's Modified Dulbecco's Medium (IMDM Sigma-Aldrich, St. Louis USA). Cell lines were purchased from the American Type Culture Collection (ATCC) and cultured as previously reported [29].

### Antibodies and human recombinant proteins

The anti-PD-L1 antibodies (PD-L1_1 and 10_12), anti-PD-1 antibody (PD-1_1), anti-LAG-3 antibody (LAG3_1) and human IgG control (unrelated) were purified as previously described [24–26]. Tb535H tribody was prepared as described in the publication number WO/2016/097408 [23]. Recombinant human PD-L1/Fc, PD-1/Fc and human LAG3/Fc proteins were purchased from Bio-Techne R&D Systems, Inc. (Northeast, Minneapolis USA). Human LAG-3/His-GST and Human HLA class II histocompatibility antigen, DRA were purchased from Cusabio Technology LLC (Houston, Texas USA). Human 5T4/His was purchased from ACROBiosystems TPG- (Newark, Delaware USA), human CD3 epsilon and CD3 delta heterodimeric protein was purchased from AMSBIO (Milton, Abingdon UK).

### Production and purification of novel 53X tribodies

Expression plasmids encoding H-chain derivatives in combination with the expression plasmid encoding L-chain derivatives for each tribody (53D, 53G, 53L1, 53L10 and 53P) were transiently transfected and expressed in Expi293 cells to produce heterodimers. After 5 days post-transfection, each tribody was purified from cell culture supernatants by His-tag affinity chromatography followed by gel filtration chromatography. This process recovers the Tribody L/Fd chain heterodimer while limiting impurities such as product variants [21, 22, 30]. The purity of the tribodies was analyzed by SDS-PAGE, and size-exclusion chromatography (SEC) analysis was performed to evaluate the tribody heterodimer purity and freeze–thaw stability. Levels of endotoxins were tested by a LAL based method. Tribodies were sterilized by 0.2 µm filtration, aliquoted and stored at -80 °C until use.

### Binding assays (ELISA)

To confirm the binding specificity of the novel generated tribodies, ELISA assays were performed on human chimeric recombinant target proteins (5T4/His, CD3δ/ε, PD-L1/Fc, PD-1/Fc or LAG-3/Fc coated at 1–5 µg/mL on 96-well plates).

The ELISA assays on coated recombinant target proteins were performed by blocking the coated plates with 5% nonfat dry milk in PBS for 1 h at 37 °C, the purified tribodies were added at different concentrations to the plates in 3% Bovine Serum Albumin (BSA Sigma St. Louis, Missouri USA) in PBS and incubated for about 90 min at room temperature by gently shaking. After extensive washes with PBS, the plates were incubated with HRP-conjugated anti-human Ig kappa antibody (Southern Biotech, North Melbourne, Victoria Australia) or with anti-His-HRP-conjugated antibody (MBL, Nowon-Ku, Seoul Korea) for 1 h, washed again and incubated with TMB reagent (Sigma-Aldrich, St Louis, Missouri USA) for 10 min before quenching with an equal volume of 1 N HCl.

Cell ELISA assays were performed by incubating the lymphocytes after stimulation with SEB (50 ng/mL for 72 h), or the HuT 78 cells, with increasing concentrations of the purified compounds in 3% BSA in PBS 1X for about 90 min at room temperature with gentle agitation. Plates were then centrifuged; cell pellets were washed with PBS and incubated with the secondary antibodies, as mentioned above.

Absorbance at 450 nm was measured by an Envision plate reader (Perkin Elmer 2102 Waltham, Massachusetts, USA).

### Competitive ELISA assays

In order to investigate the ability of the novel generated tribodies, in comparison with the corresponding parental mAbs, to compete in the PD-L1/PD-1 or LAG-3/MHC II (HLA-DRA) binding, competitive ELISA assays were performed by testing the binding of each biotinylated chimeric protein (PD-L1/Fc or MHCII) to PD-1 or LAG-3/GST-His, respectively, in the absence or in the presence of the unlabelled competitive tribodies. To this aim, NuncTM flat-bottom 96-well plate were coated with PD-1 or LAG-3 proteins. Then, the coated plates were pre-incubated with the unlabelled anti-PD-L1, anti-PD-1 or anti-LAG-3 antibodies at saturating concentrations for 2 h at room temperature (5:1 for anti-PD-L1 or -PD-1 and 3:1 for LAG-3 M/M excess ratio), and then further treated with the biotinylated PD-L1 or MHCII chimeric proteins, which were added to the plate at the same concentrations of the competitive antibodies for 2 h at room temperature. For the detection of bound biotinylated proteins, HRP-conjugated Streptavidin (Biorad, Segrate, Milano Italy) was added to the plate for 30 min and then the absorbance measured as mentioned above.

### Cell viability and cytotoxicity assays (LDH detection)

Human Peripheral Blood Mononuclear Cells (hPBMCs) were isolated from blood of healthy donors by using Greiner Leucosep® tube (Sigma-Aldrich, St Louis, Missouri USA) and cryopreserved by following the manufacturer’s instructions. Cryopreserved cell vials were thawed out, collected for resting and counted before use, as previously reported [30].

To measure the cytotoxic effects of the novel generated tribodies, target tumor cells were co-cultured with hPBMCs (effector: target ratio 5:1) and treated with the tribodies used at increasing concentrations, or with the parental mAbs and their corresponding combinations used in parallel assays at the same concentrations. To this aim, tumor cells were plated in 96-well flat-bottom plates at the density of 1 × 10^4^ cells/well, for 16 h. Then, hPBMCs isolated from healthy donors were added in the absence or in the presence of the tribodies or antibodies and incubated at 37 °C for 48 h. Untreated cells or cells treated with an unrelated human IgG_4_ were used as negative controls.

The lysis of the target cells was measured by detecting the levels of LDH released in the supernatant of the co-cultures, by using the LDH detection kit (Thermofisher Scientific, Rockford, Illinois USA), following the manufacturer’s recommendations. Cell lysis was analyzed by measuring the fold increase of LDH in the presence of each treatment, with respect to the amount present in the supernatant of co-cultures untreated or treated with the unrelated antibodies and expressed as percentage with respect to the maximum lysis represented by the cells treated with 10% triton.

### Cytokine secretion assays

The secretion of human IL-2 and IFNγ in supernatants of co-cultures of tumor cells with hPBMCs, or in the supernatants of hPBMCs treated with the tribodies or their corresponding parental mAbs, was evaluated by DuoSet ELISA kit assays (Bio-Thecne R&D Systems, Inc. Northeast, Minneapolis USA). Briefly, after treatments culture supernatants were centrifuged and the cytokines quantified by using the IL-2 and IFNγ kits (from Bio-Thecne R&D Systems, Inc. Northeast, Minneapolis USA), according to the producer’s recommendations. Concentration values were converted in pg/mL and reported as the mean of at least three determinations.

### T cell activation bioassays

TCR/CD3 activation in the presence of 5T4-expressing cells was performed by T cell activation bioassay (NFAT) (Promega, Madison, Wisconsin USA) in accordance with manufacturer’s instructions. Briefly, genetically engineered Jurkat T cell line which expresses a luciferase reporter driven by a NFAT-response element (included in the kit) were co-cultured with 5T4-expressing CHO-K1 cells in the presence of increasing concentrations of parental Tb535H or novel 53X tribodies. After 4 h incubation at 37 ℃, Bio-Glo™ reagent (included in the kit) was added and luminescence was measured by a luminometer.

### In vivo efficacy studies

Five to 6 weeks female NOD/ShiJic-scidJcl mice (NOD-SCID) (Japan CLEA) were used for efficacy studies. A-549 human alveolar lung adenocarcinoma cells were harvested from cell culture dishes by trypsinization, washed with PBS and prepared as cell suspension. Human PBMCs (Cellular Technology Limited) were cultured in vitro in the presence of Dynabeads human T-activator (CD3/CD28) (Veritas Labettor B.V. Groningen, Netherlands EU) for 4 days before inoculation in mice with A-549 cells. Cell suspensions of 5 × 10^6^ A-549 cells were mixed with equal number of activated hPBMCs (the ratio of implanted activated hPBMCs to A-549 cancer cells was 1:1). Mixed cell suspensions were subcutaneously transplanted in right flank of NOD-SCID mice (day 0) and intravenous treatments of indicated tribodies at the indicated dosage were performed at day 0, 2, 4, 6, 8, 10 (total 6 treatment) after the cell transplantation. Tumor growth was measured with calipers in 2 perpendicular dimensions, and tumor volume (mm^3^) was calculated by using the formula (width^2^ x length) x π/6. Tumor volumes were expressed as mean ± standard deviation (SD). Statistical significances of tumor size differences on the final day of each study were evaluated by Dunnett test (vs vehicle treatment group), and *P* < 0.05 was mentioned as significant difference.

### Statistical analyses

Error bars were calculated on the basis of the results obtained by at least three independent experiments. Statistical analyses of in vitro experiments were assessed by Student’s t-test (two variables), and statistical significance was established as *** *P* ≤ 0.001; ** *P* < 0.01; * *P* < 0.05. Statistical analyses of tumor size differences of each in vivo study were evaluated by Dunnett test (vs vehicle treatment group), and statistical significance was established as *P* < 0.05.

## Results

### In vitro effects of combinatorial treatments of Tb535H with immunomodulatory mAbs on the stimulation of hPBMCs

We firstly investigated on novel combinations of multiple immunomodulatory mAbs, recently isolated in our laboratory, specific for PD-L1, PD-1 or LAG-3 and able to strong activate T cells [24–28], with the parental bi-specific tribody (Tb535H) targeting the 5T4-TAA on tumor cells and CD3 on T cells, which already demonstrated anti-tumor efficacy in preclinical studies [23]. These novel combinations were tested on PD-L1-positive MDA-MB 231 and BT-549 breast cancer cells expressing 5T4, co-cultured for 48 h with lymphocytes (Effector:Target cells ratio 5:1), by using the Tb535H tribody (1 nM) plus each of the immunomodulatory mAbs (50 nM) in comparison with each single parental compound (Supplementary Fig. 1A and B). Cell surface expression of 5T4 and PD-L1 on these cells were confirmed by flow cytometric analyses (data not shown). Interestingly, we found that the addition of the anti-PD-L1, -PD-1 or LAG-3 mAb to the tribody significantly increased the tumor cells lysis accordingly to the higher secretion of IFNγ cytokine, detected after treatments (Supplementary Fig. 1). In parallel, the same combinations tested on PD-L1, 5T4-negative MCF-7 cancer cells showed no significant increase of these effects, thus confirming the specificity of the combinatorial treatments (Supplementary Fig. 1C).

### Construction and purification of novel 53X tribodies

**Fig. 1:**
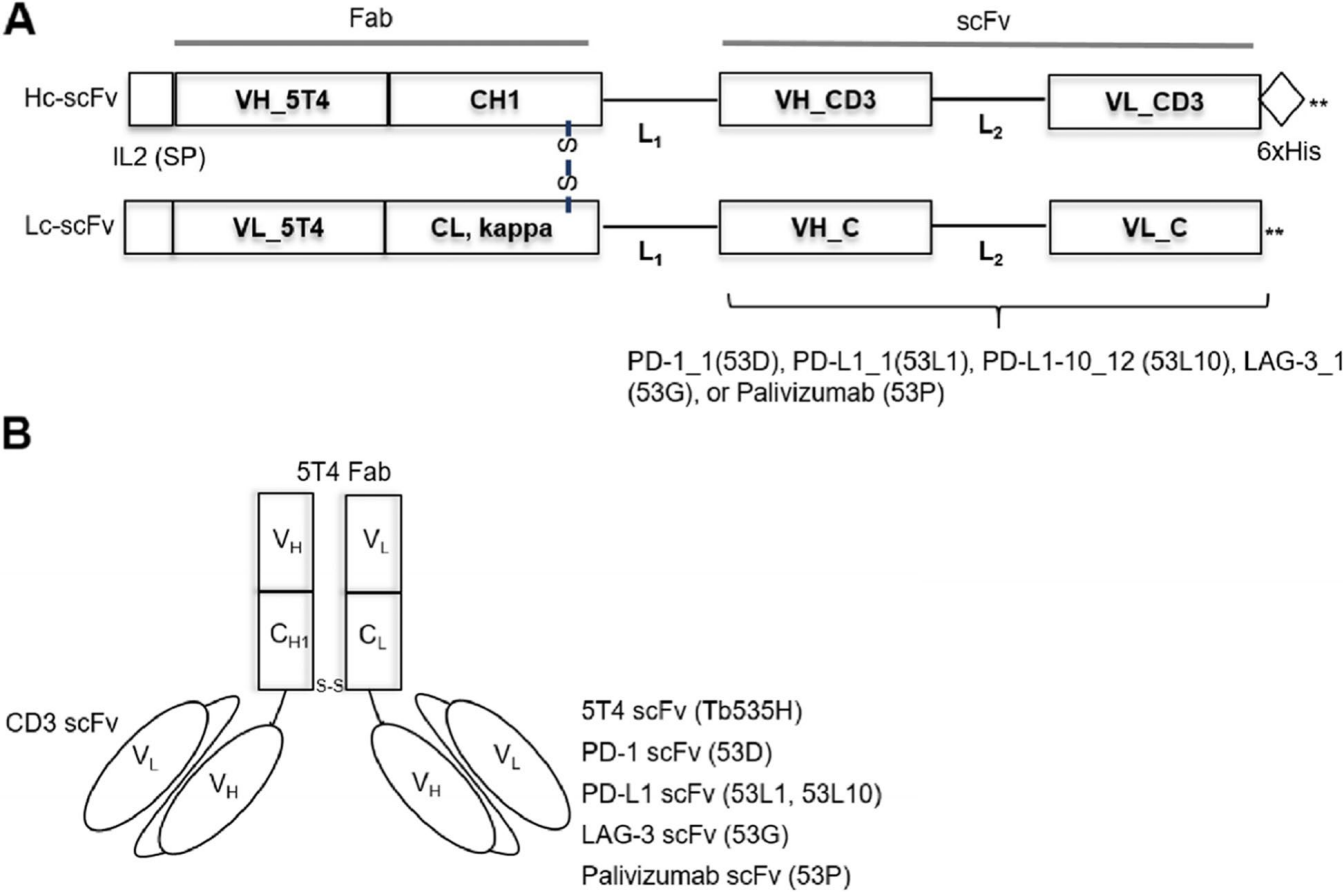
Schematical representation of 53X tribodies. **A** Scheme of constructs for 53X tribodies. SP, human interleukin (IL-2) signal peptide; VH_5T4, VL_5T4, amino acid sequences coding for the immunoglobulin heavy and light chain variable regions from Tb535H with specificity for human 5T4; CH1, CL, amino acid sequences coding for the human immunoglobulin heavy chain constant region 1 and the kappa light chain constant region, respectively; VH_CD3, VL_CD3, amino acid sequences coding for the variable heavy and light chain regions from humanized OKT3 building a scFv with specificity for human CD3; L1, L2, aminoacid sequences with flexible linker, GPGGGSPG, and GGGGSGGGGSGGGGS [(GGGS)_3_], respectively. VH_C, VL_C, amino acid sequences coding for the variable heavy and light chain from PD-1_1, PD-L1_1, 10_12, LAG3_1, scFvs with specificities for human PD-1, PD-L1 or LAG-3, respectively. The scFv from Palivizumab was used in an additional tribody for isotype control; 6xHis, amino acid sequences coding for a hexahistidine tag. **B** Scheme of assembled tribody proteins for Tb535H [(5T4)_2_ × CD3], 53D [5T4 x CD3 x PD-1], 53L1 [5T4 x CD3 x PD-L1], 53L10 [5T4 x CD3 x PD-L1], 53G [5T4 x CD3 x LAG-3] and 53P [5T4 x CD3 x isotype control], respectively

The promising results of the combined treatments prompted us to generate five novel tri-specific tribodies, as shown in Fig. 1A and B and listed in Table 1. The tribody is a multi-specific antibody construct made up of a Fab domain as a scaffold, in which Fd fragment of H-chain (V_H_ + CH_1_) and L-chain (V_L_ + C_L_) can naturally heterodimerize in vivo, and additional functions, such as scFvs, can be incorporated on the scaffold. Parental Tb535H is a bi-specific tribody, endowed with a bivalent binding for 5T4 through its Fab and one of scFv domains, and a monovalent CD3 binding through another scFv [23]. The CD3 binding scFv used both in the parental Tb535H and novel 53X tribodies was generated from OKT3 mAb, and was chosen as not able to induce the non-specific activation of T cells and cytokines secretion in the absence of tumor cells at concentrations up to 1,000 ng/mL, as previously described in the patent documents of parental Tb535H (PCT/EP2015/080795, WO2016/097408 A1). The novel 53X tribodies generated here, have binding specificity to 3 different molecules, 5T4, CD3 and an IC, such as PD-L1, PD-1, or LAG3, in a single molecule. Indeed, the 5T4 binding scFv arm of Tb535H [23] was replaced by a binding moiety derived from PD-1_1, PD-L1_1, 10_12, LAG3_1 mAbs [24, 25]. The mAb 10_12 is an affinity-matured variant of PD-L1_1 [24], whereas Palivizumab [31] was used as a negative control. The novel tribodies were named as 53D [(Fab)5T4 x (scFv) CD3 x (scFv) PD-1(PD-1_1)], 53L1 [(Fab)5T4 x (scFv) CD3 x (scFv) PD-L1(PD-L1_1)], 53L10 [(Fab)5T4 x (scFv) CD3 x (scFv) PD-L1(10_12)], 53G [(Fab)5T4 x (scFv) CD3 x (scFv) LAG3(LAG3_1)], 53P [(Fab)5T4 x (scFv) CD3 x (scFv) Palivizumab], respectively. The latter one was constructed as an isotype control for the other 53X tribodies. All the tribodies were expressed and purified as described in Methods, and their purity and stability was checked by SDS-PAGE and size-exclusion chromatography (data not shown).

**Table 1:** List of tribodies generated in the study

Name of product	Structure		Fab	scFv
Tb535H	(Fab) 5T4 – (scFv) CD3 – (scFv) 5T4	H – chain derivative	hu5T4	huOKT3
		L – chain derivative	hu5T4	hu5T4
53L1	(Fab) 5T4 – (scFv) CD3 – (scFv) PD-L1	H – chain derivative	hu5T4	huOKT3
		L – chain derivative	hu5T4	PD-L1_1
53L10	(Fab) 5T4 – (scFv) CD3 – (scFv) PD-L1	H – chain derivative	hu5T4	huOKT3
		L – chain derivative	hu5T4	10_12
53D	(Fab) 5T4 – (scFv) CD3 – (scFv) PD-1	H – chain derivative	hu5T4	huOKT3
		L – chain derivative	hu5T4	PD-1_1
53G	(Fab) 5T4 – (scFv) CD3 – (scFv) LAG-3	H – chain derivative	hu5T4	huOKT3
		L – chain derivative	hu5T4	LAG-3_1
53P	(Fab) 5T4 – (scFv) CD3 – (scFv) Palivizumab	H – chain derivative	hu5T4	huOKT3
		L – chain derivative	hu5T4	Palivizumab

Amino acidic sequences of Tb535H were described in WO2016097408 (A1). Amino acidic sequences of PD-1_1, PD-L1_1, 10_12, LAG-3_1 were described in WO2019180201A2

### Binding affinity of 53X tribodies for recombinant 5T4 and CD3 proteins by Enzyme-Linked Immuno Sorbent Assays (ELISA)

**Fig. 2:**
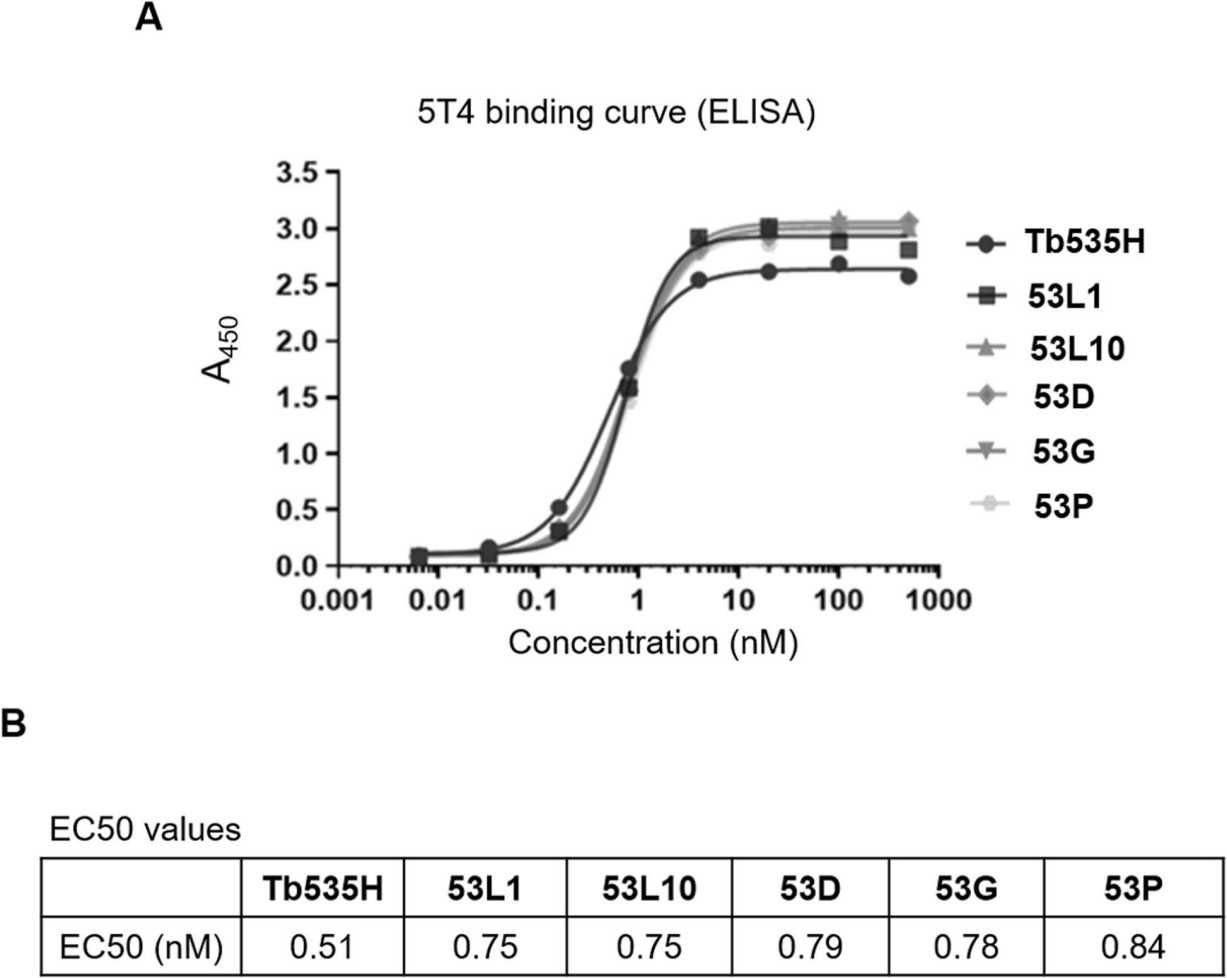
Binding affinity of parental Tb535H and novel 53X tribodies to recombinant 5T4 protein by ELISA. **A** Binding curves by ELISA assays of tribodies (0–500 nM) to immobilized recombinant human 5T4 protein. Binding values were reported as the mean of determinations obtained in three independent experiments. **B** Table reporting the binding affinity or EC50 (nM) values for 5T4 of each indicated construct. Standard Deviations were ≤ 3—10%

To verify whether the novel generated tri-specific 53X tribodies 53D, 53L1, 53L10, 53G and 53P retain the ability of parental Tb535H to bind to 5T4 antigen, binding affinity of purified tribodies to 5T4 protein was examined by ELISA (Fig. 2). Recombinant human 5T4 protein was immobilized onto 96-well ELISA plates. The binding assays of parental Tb535H and purified novel tribodies were performed by testing them at increasing concentrations (0 – 500 nM) according to the procedure described in Materials and Methods. As shown in Fig. 2A, all the constructed tribodies showed concentration dependent binding to 5T4 with similar or even better EC50 (nM) values than that of the parental Tb535H (Fig. 2B). Even though the parental Tb535H has two 5T4 binding arms derived from a Fab and a scFv, differently from the novel constructed 53X tribodies that have a monovalent 5T4 binding arm derived from a single Fab, the results indicated that the novel tribodies show a comparable or better binding affinity (Kd), thus demonstrating that a single anti-5T4 arm (Fab) is sufficient for retaining similar 5T4 binding of the parental Tb535H in all the novel 53X tribodies.

**Fig. 3:**
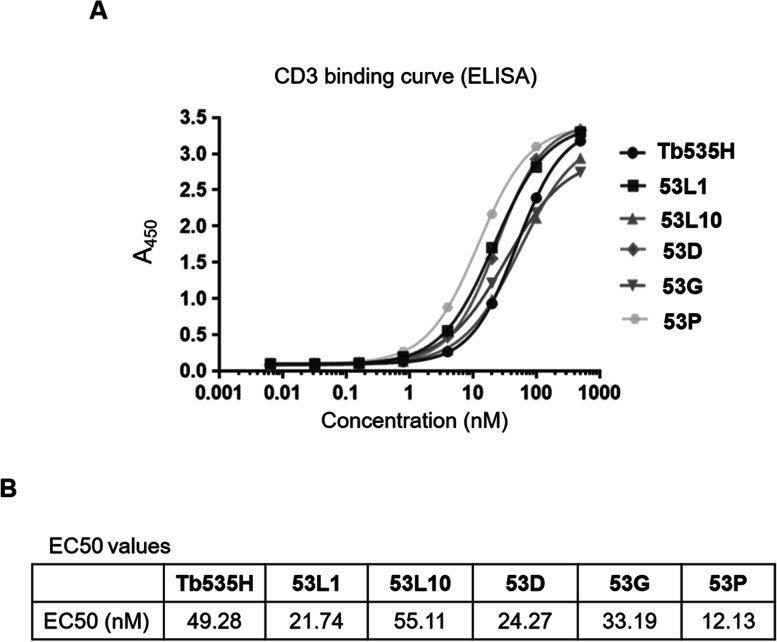
Binding affinity of parental Tb535H and novel 53X tribodies to recombinant CD3 protein by ELISA. **A** Binding curves by ELISA assays of tribodies (0 – 500 nM) to immobilized recombinant human CD3ε/δ heterodimer. Binding values were reported as the mean of determinations obtained in three independent experiments. **B** Table reporting the EC50 (nM) values for CD3 binding of each indicated tribody. Standard Deviations were ≤ 3—10%

To verify whether the constructed novel 53X tribodies 53D, 53L1, 53L10, 53G and 53P, retain the ability of the parental Tb535H to bind to CD3 antigen, binding affinity of purified tribodies to recombinant CD3 protein was examined by ELISA (Fig. 3). Recombinant human CD3δ/ε heterodimeric protein was immobilized onto 96-well ELISA plates. The binding assays of Tb535H and purified novel tribodies tested at increasing concentrations (0 – 500 nM) were carried out according to the procedure described in Materials and Methods. As shown in Fig. 3A, all constructed tribodies showed concentration dependent CD3 binding with similar EC50 (nM) values (Fig. 3B). The results suggest that the novel tribodies fully retain the ability of parental Tb535H to bind to human CD3 protein.

### Binding by ELISA of novel 53X tribodies to recombinant IC proteins and activated hPBMCs

**Fig. 4:**
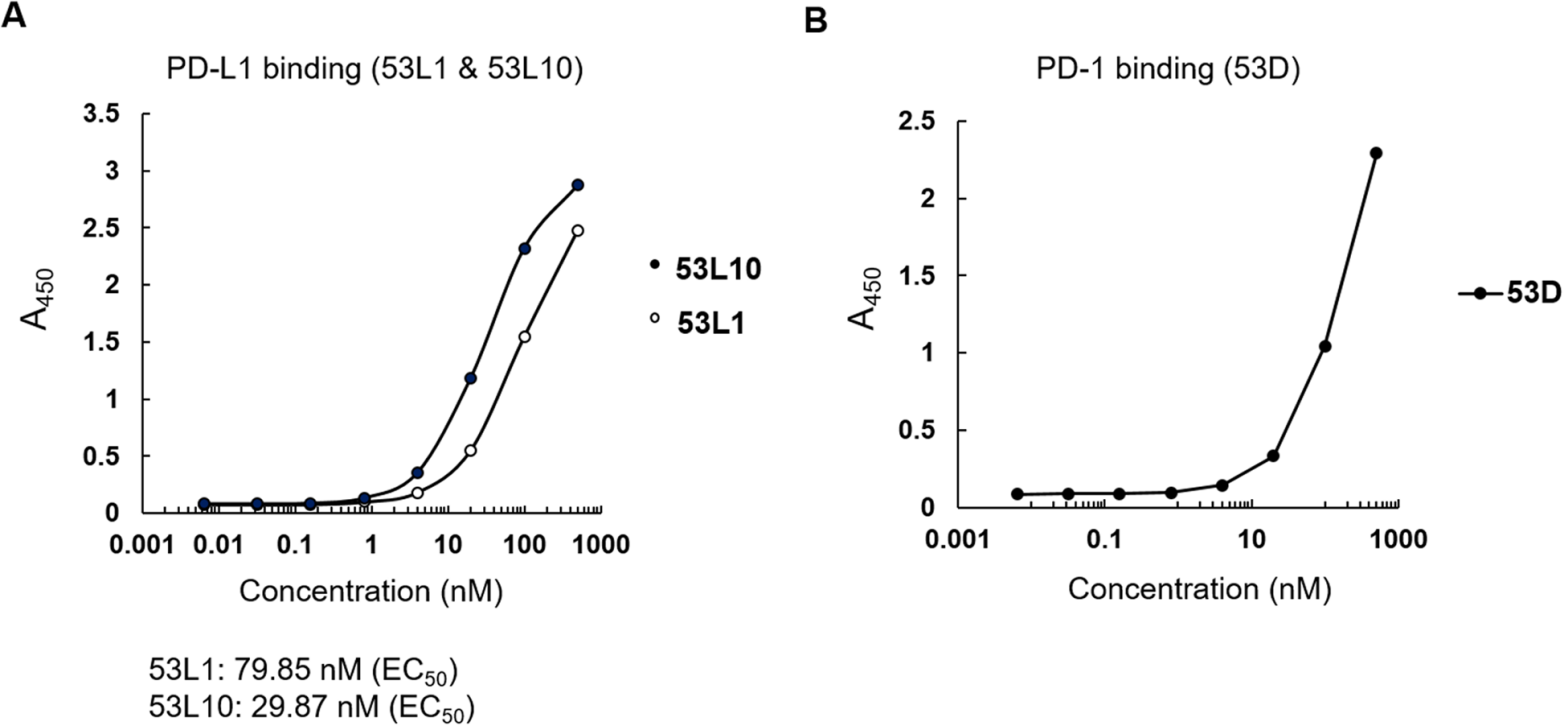
Binding affinity of indicated tribodies for recombinant PD-L1 or PD-1 proteins by ELISA. **A** Binding curves obtained by ELISA assays of 53L1 and 53L10 tribodies (0 – 500 nM) on immobilized recombinant human PD-L1 proteins. **B** Binding curves obtained by ELISA of 53D tribody (0–500 nM) tested on immobilized recombinant human PD-1 protein. Binding values were reported as the mean of at least three determinations obtained in three independent experiments. Standard Deviations were ≤ 3—10%

To verify whether the novel tribodies including a binding arm to PD-L1 or PD-1 have a satisfactory binding affinity for their target recombinant proteins, we performed ELISA assays on immobilized recombinant PD-1 or PD-L1 proteins (Fig. 4). Tribodies 53L1 and 53L10, which have a binding arm specific for PD-L1, showed binding to recombinant PD-L1 protein in a concentration dependent manner with EC50 values of 79.85 nM and 29.87 nM, respectively (Fig. 4A). The tribody 53D, which contains a binding arm to PD-1, also showed a concentration-dependent binding to recombinant PD-1 protein (Fig. 4B).

The Kd values obtained on PD-1 and PD-L1 for the tribodies are comparable to those of their parental mAbs even though the maximum absorbance value was found to be lower.

These results were confirmed when the novel 53X tribodies were tested by cell ELISA assays (Supplementary Fig. 2) for their binding to the target proteins on SEB (Staphylococcal enterotoxin B) activated lymphocytes expressing CD3 and the target ICs in their native conformation.

All the novel tribodies 53D, 53L1, 53L10, 53G were found able to bind to activated hPBMCs with a higher affinity than the parental Tb535H lacking the IC binding moiety (Supplementary Fig. 2).

To further confirm the binding specificity of the tribody 53G for LAG-3, we investigated also its binding on HuT 78 cell line, which is derived from CD4^+^ human T cell lymphoma and expresses high levels of LAG-3 receptor, but low or absent levels of PD-1 and PD-L1. We tested it at increasing concentrations, in parallel with the LAG-3_1 parental mAb, by cell ELISA and we found that the tribody 53G bound to the cells with a comparable specificity (Supplementary Fig. 3).

### Antagonistic effects of the 53X tribodies tested by competitive ELISA assays

**Fig. 5:**
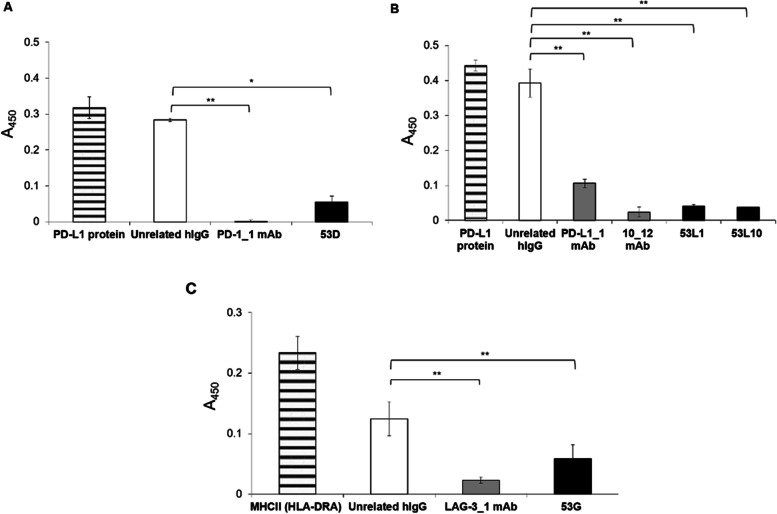
Antagonistic effects of the 53X tribodies tested by Competitive ELISA assays. The ELISA assays were performed by measuring the binding of each biotinylated chimeric PD-L1 or MHC II protein (striped bars) to immobilized PD-1 (**A-B**) or LAG-3 (**C**) proteins, respectively, in the absence or in the presence of the unlabelled competitive anti-PD-L1 (53L1 and 53L10) (**A**), anti-PD-1 (53D) (**B**) or anti-LAG-3 (53G) (**C**) tribodies (black bars) or their respective parental mAbs (grey bars) used at saturating concentrations (5:1 M/M for anti-PD-L1 or anti-PD-1, and 3:1 M/M for anti-LAG-3 excess ratio). Unrelated IgGs were used as negative controls (empty bars). Binding values were reported as the mean of at least three determinations obtained in three independent experiments. Error bars depicted means ± SD. *P*-values for the indicated compounds are: ** *P* < 0.01; * *P* < 0.05 by student’s t-test (two variables)

To verify whether the novel human tribodies 53D, 53L1 and 53L10, derived from PD-1_1, PD-L1_1 and 10_12 antibody binding domains, respectively, retain the ability of the parental mAbs to interfere in PD-1/PD-L1 interaction, we performed competitive ELISA assays by measuring the binding of biotinylated PD-L1 ligand to immobilized PD-1 receptor in the absence or in the presence of a molar excess (5:1 M/M) of the tribodies 53D, 53L1, 53L10 or their parental mAbs PD-L1_1,PD-1_1 and 10_12, used in parallel assays as positive controls. An unrelated human IgG was used as negative control. As reported in Fig. 5A and B, the binding of biotinylated PD-L1 ligand was significantly reduced in the presence of the tribodies with respect to the binding signal of the biotinylated PD-L1 used alone, thus suggesting that the novel tribodies retain the ability of the parental mAbs to interfere in the interactions of PD-L1 with PD-1.

In parallel assays, we analyzed the ability of the tribody 53G, to interfere in LAG-3/MHCII (HLA-DRA) interaction, by performing similar competitive ELISA assays. To this aim, LAG-3-His-GST recombinant protein was coated on plates at the concentration of 50 nM, the tribody 53G or its parental mAb LAG-3_1 were added at saturating concentration of 2 μM and the binding of the Biotinylated-MHCII protein (700 nM) was measured. As shown in Fig. 5C, the binding of biotinylated MHCII was strongly reduced in the presence of the tribody 53G or the parental LAG-3_1 mAb, used as control, with respect to the binding signal of the biotinylated MHC II used alone.

### T cell activation bioassays in the presence of 5T4-expressing target cells

**Fig. 6:**
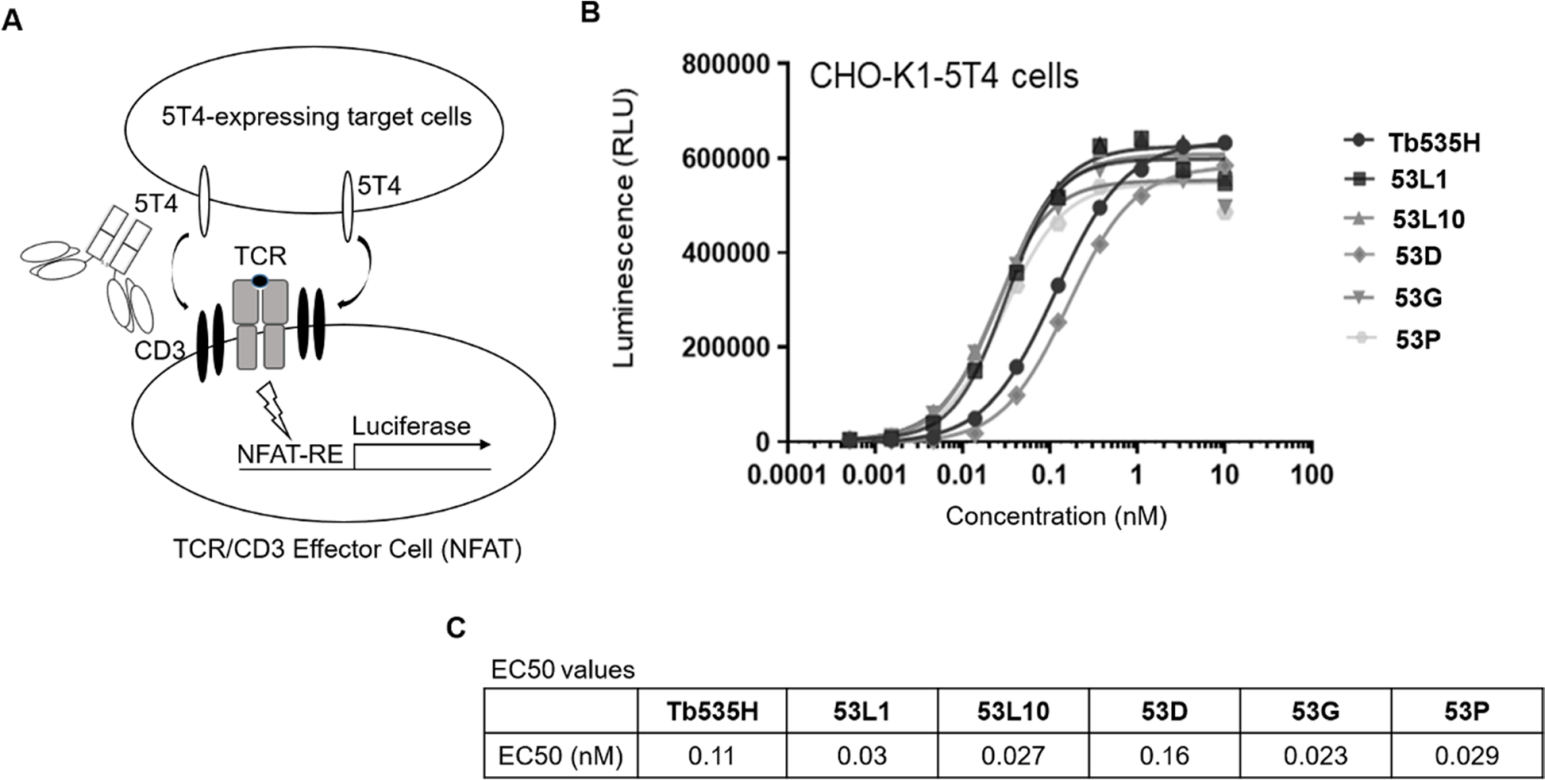
T cell activation bioassays in the presence of 5T4-expressing target cells. **A** Schematic representation of TCR/CD3 activation in the presence of 5T4-expressing cells by T cell activation bioassay (NFAT). **B** Genetically engineered Jurkat T cells which expresses a luciferase reporter driven by a NFAT-response element (NFAT-RE) (“TCR/CD3 effector cells”) were incubated with increasing concentrations of the indicated tribodies in the presence of CHO-K1-5T4 cells. After 4 h incubation at 37℃, Bio-Glo™ reagent was added and luminescence was measured by a luminometer. Luminecence values were reported as the mean of determinations obtained in three independent experiments. **C** Table reporting the EC50 (nM) values for 5T4-mediated TCR/CD3 activation of each indicated construct

Activation of T cell receptor (TCR)/CD3 on effector cells by novel tribodies and parental Tb535H was examined on 5T4-expressing CHO-K1 (CHO-5T4) cells, used as target cells. The assays were performed by using T cell activation bioassays (NFAT). Figure 6A shows the schematic representation of the assay. When cell surface 5T4 on target cells and CD3 on the effector cells are cross-linked with a bi-specific T cell engager, TCR/CD3 transduces intracellular signals resulting in NFAT-response element (NFAT-RE)-mediated luminescence. Genetically engineered Jurkat T cell line which expresses a luciferase reporter driven by a NFAT-RE (“TCR/CD3 effector cells”) were co-cultured with CHO-5T4 cells (“target cells”) in the presence of increasing concentrations of parental Tb535H or novel 53X tribodies, as indicated (Fig. 6B). Parental Tb535H induced luciferase activity in a concentration dependent manner and EC_50_ was found to be 0.11 nM. The novel tribodies also induced a concentration-dependent luciferase activity with EC_50_ values in the range of 0.03 nM – 0.16 nM (Fig. 6C). The 5T4-mediated CD3 activation by novel tribodies (with the exception of 53D) was slightly better than that of the parental Tb535H; the EC_50_ values of novel tribodies were within almost three-fold lower than that of parental Tb535H.

### In vitro cytotoxic effects of the novel human tribodies on human tumor cells co-cultured with human lymphocytes

**Fig. 7:**
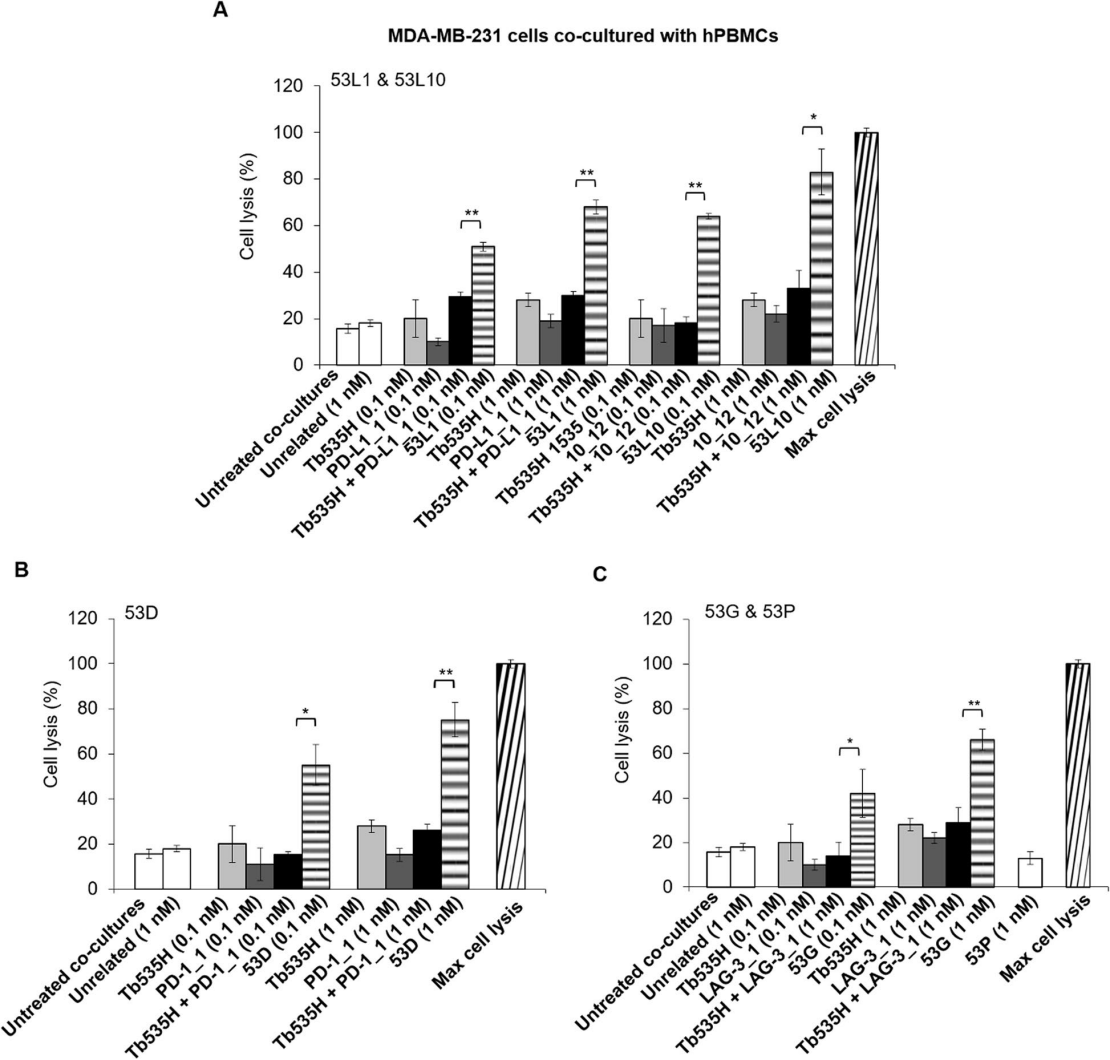
Cytotoxic effects of the novel tribodies or the combinations of Tb535H with the parental mAbs on MDA-MB-231 cells co-cultured with hPBMCs. Breast MDA-MB-231 tumor cells were co-cultured with hPBMCs (Effector:Target cells ratio 5:1) and treated for 48 h with (**A**) 53L1 and 53L10 (striped bars), (**B**) 53D (striped bars), (**C**) 53G (striped bars). Tb535H (light grey bars), parental immunomodulatory mAbs (dark grey bars) or their combinations (black bars) were also tested at the indicated concentrations. Co-cultures untreated or treated with the unrelated Palivizumab-derived tribody 53P (**C**, empty bars) were used as negative controls. Cell lysis was measured by detecting LDH release, as described in Methods. Error bars depict means ± SD. *P*-values for the indicated compounds are: *** *P* ≤ 0,001; ** *P* < 0.01; * *P* < 0.05 by student’s t-test (two variables)

**Fig. 8:**
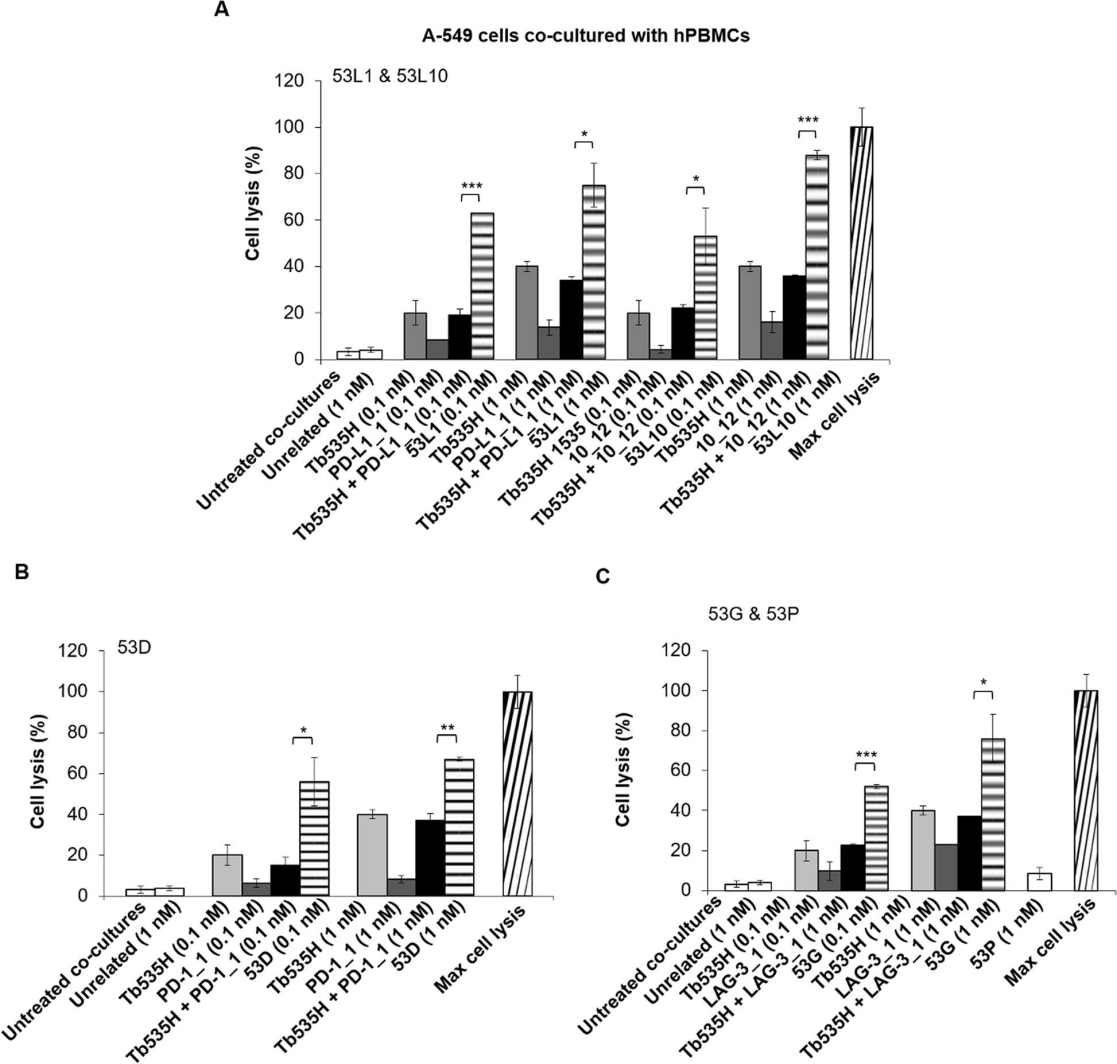
Cytotoxic effects of the novel tribodies or the combinations of Tb535H with the parental mAbs on A-549 cells co-cultured with hPBMCs. Lung A-549 tumor cells were co-cultured with hPBMCs (Effector:Target cells ratio 5:1) and treated for 48 h with (**A**) 53L1 and 53L10 (striped bars), (**B**) 53D (striped bars) or (**C**) 53G (striped bars). Tb535H (light grey bars), parental immunomodulatory mAbs (dark grey bars) or their combinations (black bars) were also tested in parallel assays at the indicated concentrations. Co-cultures untreated or treated with the unrelated tribody 53P (**C**, empty bars) were used as negative controls. Cell lysis was measured by detecting LDH release, as described in Methods. Error bars depict means ± SD. *P*-values for the indicated compounds are: *** *P* ≤ 0,001; ** *P* < 0.01; * *P* < 0.05 by student’s t-test (two variables)

To verify whether the novel tribodies targeting PD-L1, PD-1 and LAG-3, efficiently induce the activation of lymphocytes against cancer cells, we investigated their effects on co-cultures of tumor cells and lymphocytes. To this aim, MDA-MB-231 breast cancer cells or lung A-549 cancer cells expressing high levels of PD-L1 and 5T4 were co-cultured with hPBMCs (Effector: Target ratio of 5:1) in the absence or presence of increasing concentrations of the tribodies 53D, 53G, 53L1 and 53L10 for 48 h at 37°C. In parallel assays, we tested their parental mAbs PD-L1_1, 10_12, PD-1_1 and LAG-3_1 used in combination with the parental tribody Tb535H at the same concentrations and a control tribody (53P), derived from Tb535H but lacking immunomodulatory antibody fragments. After the incubation, cells lysis was measured by the lactate dehydrogenase (LDH) released in the cell supernatants by using LDH assays**.** The results show that all the novel tribodies, with the exception of 53P, used as a negative control, induced tumor cells lysis with higher efficacy than the parental mAbs or their combinations with Tb535H (see Figs. 7 and 8). Specifically, all the tribodies at least doubled the levels of LDH release of their corresponding parental mAbs and parental Tb535H tribody used in combination, with the tribodies 53L1 and 53L10 reaching about 80–90% of lysis at a concentration of 1 nM.

**Fig. 9:**
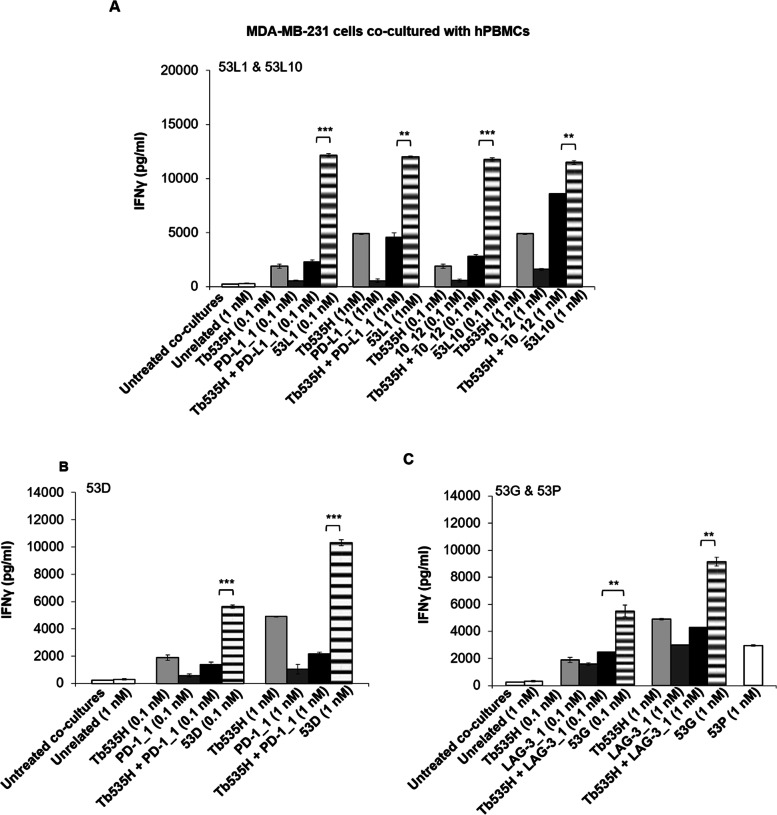
Effects of novel tribodies or the combinations of Tb535H with the parental mAbs on the secretion of IFNγ by co-cultures of MDA-MB-231 with hPBMCs. The secretion of IFNγ was measured by ELISA on supernatants of MDA-MB-231 cells co-cultured with hPBMCs and treated for 48 h with (**A**) 53L1 and 53L10 (striped bars), (**B**) 53D (striped bars), (**C**) 53G (striped bars). Tb535H (light grey bars), parental immunomodulatory mAbs (dark grey bars) or their combinations (black bars) were also tested at the indicated concentrations for comparison. Co-cultures untreated or treated with the unrelated tribody 53P (**C**, empty bars) were used as negative controls. Error bars depict means ± SD. *P*-values for the indicated compounds are: *** *P* ≤ 0,001; ** *P* < 0.01; by student’s t-test (two variables)

**Fig. 10:**
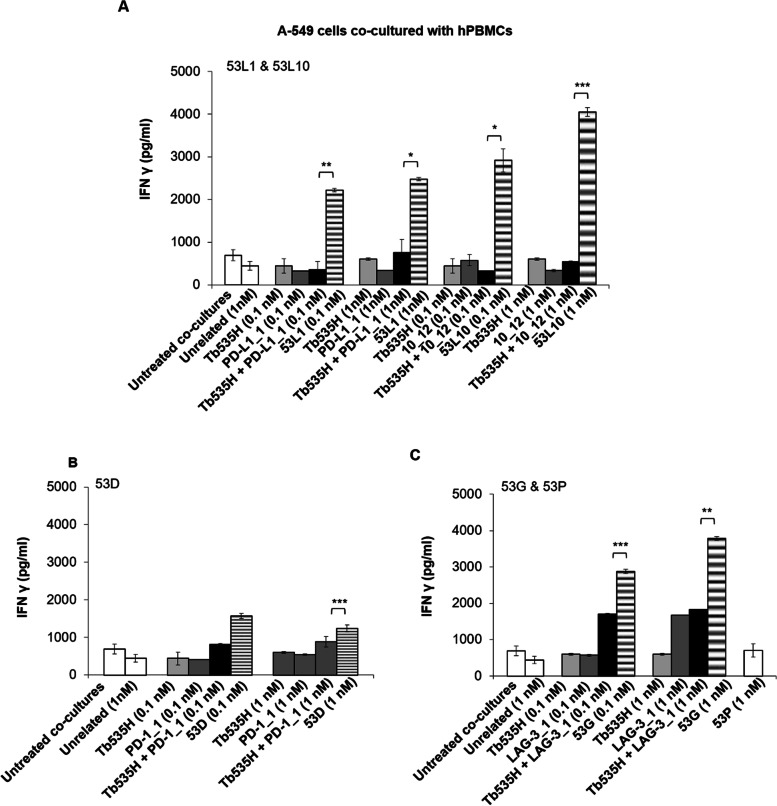
Effects of novel tribodies or the combinations of Tb535H with the parental mAbs on the secretion of IFNγ by co-cultures of A-549 cells with hPBMCs. The secretion of IFNγ was measured by ELISA on supernatants of A-549 cells co-cultured with hPBMCs and treated for 48 h with (**A**) 53L1 and 53L10 (striped bars), (**B**) 53D (striped bars), (**C**) 53G (striped bars). Tb535H (light grey bars), parental immunomodulatory mAbs (dark grey bars) or their combinations (black bars) were also tested at the indicated concentrations for comparison. Co-cultures untreated or treated with the unrelated tribody 53P (**C**, empty bars) were used as negative controls. Error bars depict means ± SD. *P*-values for the indicated compounds are: *** *P* ≤ 0,001; ** *P* < 0.01; * *P* < 0.05 by student’s t-test (two variables)

In order to clarify whether the enhanced tumor cell lysis mediated by the novel tribodies is correlated to the increased activation of lymphocytes, we measured the levels of IFNγ, as a marker of hPBMCs functionality [32], released in the supernatants of these co-cultures, by comparing the effects of the novel tribodies with those of the parental mAbs when used in combination with parental tribody Tb535H. As shown, IFNγ levels were much higher in the supernatants of MDA-MB-231 (Fig. 9) and A-549 (Fig. 10) co-cultures treated with the novel tribodies with respect to those observed in the treatments with their parental tribody Tb535H, their parental mAbs PD-L1_1, 10_12, PD-1_1 and LAG-3_1, or their combinations.

To exclude the possibility that the 53X tribodies exert nonspecific side effects on circulating naive lymphocytes in the absence of tumor cells, the novel tribodies were incubated with unstimulated lymphocytes for 48–66 h. The levels of IL-2 and IFNγ cytokines released from unstimulated lymphocytes treated with tribodies were found to be very low (Supplementary Fig. 4), thus proving that the effects of the tribodies are exerted only on activated lymphocytes expressing the ICs in the presence of tumor cells.

Thus, these results clearly confirm the validity of our strategy to insert an immunomodulatory moiety in the parental Tb535H to increase its anti-tumor efficacy with no significant enhancement of off-target side effects.

### In vivo anti-tumor efficacy of novel 53X tribodies

**Fig. 11:**
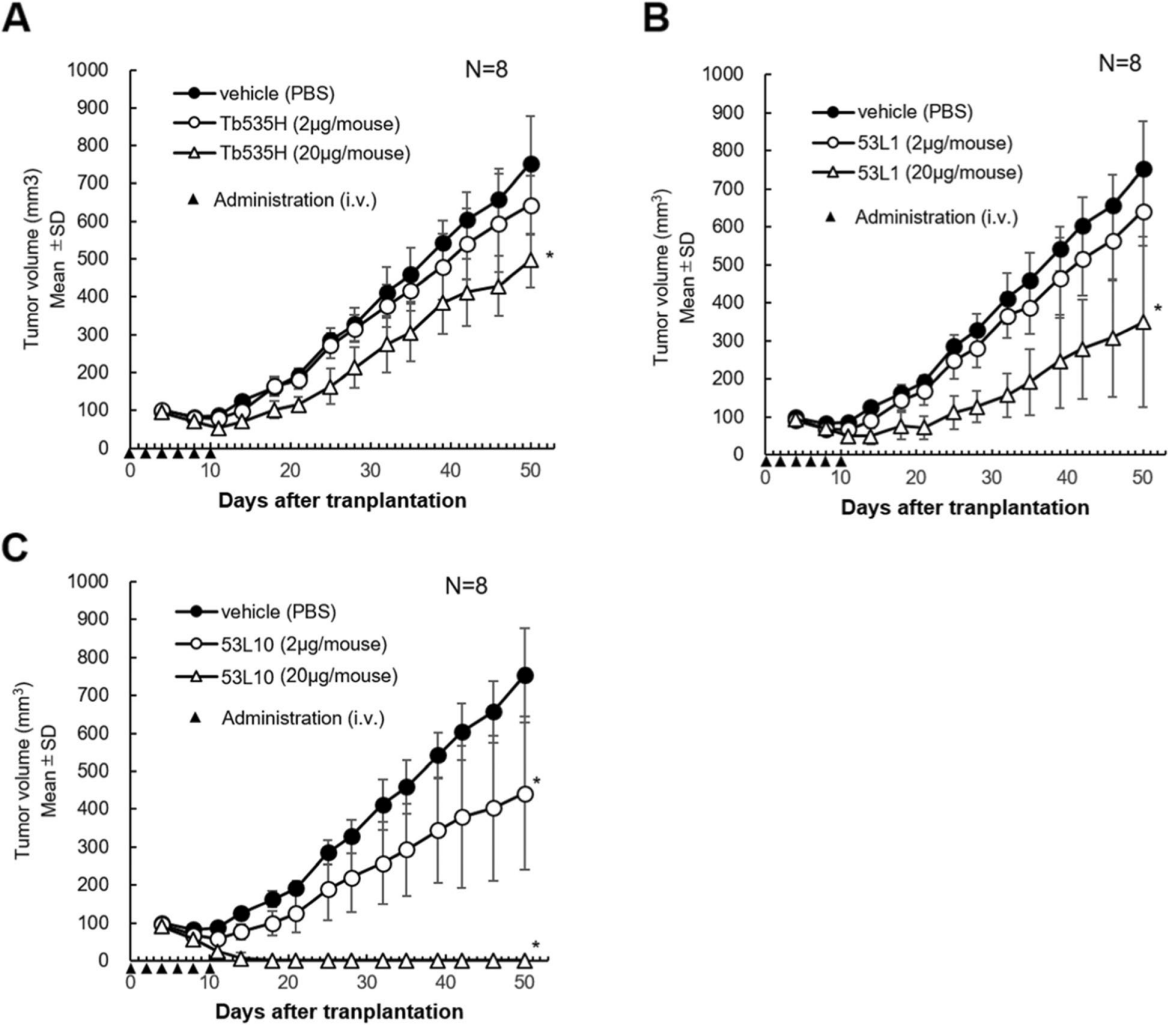
In vivo Anti-tumor efficacy of parental Tb535H, 53L1 and 53L10 novel tribodies. Tumor growth curves of A-549 subcutaneous tumors treated in the presence of human PBMCs with (**A**) parental Tb535H, (**B**) 53L1, and (**C**) 53L10 at two different doses (〇: 2 μg/mouse treatment, △: 20 μg/mouse treatment). ●: vehicle (PBS) treatment. Five × 10^6^ cells of A-549 cells were subcutaneously transplanted into right flank of NOD-scid mice together with equal number of human activated PBMC (hPBMC). hPBMC were stimulated with Dynabeads Human T-Activator CD3/CD28 (Veritas) for 4 days before the transplantation. Intravenous treatment of indicated samples at the indicated dosage were performed at day 0, 2, 4, 6, 8, 10 (total 6 treatment) after the cell transplantation. Tumor volumes were expressed as mean ± standard deviation (SD). *P < 0.05 by Dunnett test (vs vehicle treatment group)

In vivo anti-tumor efficacy of the novel tribodies was evaluated on A-549 (human lung cancer cells) xenograft mouse models. Human PBMCs were activated by Dynabeads Human T activator CD3/CD28 for 4 days before the transplantation (activated PBMCs) to amplify the T cell population responsible for cytotoxicity of Tb535H against tumor cells. A-549 lung cancer cells were subcutaneously transplanted in the right flank with equal number of activated human PBMCs in immunodeficient NOD-SCID mice (day 0). Intravenous administrations of the indicated tribodies were performed every other day from day 0 to day 10 (total 6 treatments) (Fig. 11). In the parental Tb535H treatment group, significant tumor growth inhibition was observed at the dose of 20 µg/mouse compared with vehicle treatment (*P* < 0.05), although the lower dosage (2 µg/mouse) did not show significant tumor growth inhibition (Fig. 11A). On the other hand, the anti-tumor activity of 53L1 (20 µg/mouse) was significantly potentiated compared with that of Tb535H used at the same dosage (Fig. 11B). Furthermore, in 53L10 treatment group, significant tumor growth inhibition of A-549 tumor by treatment with both 2 µg/mouse and 20 µg/mouse dosage was observed compared with vehicle treatment (Fig. 11C). The tumor growth inhibition (TGI; %) in 53L10 (2 µg/mouse) treatment group on day 50 reached 41.5% compared with that of vehicle treatment group (*P* < 0.05) and 100% TGI by 53L10 at higher dosage treatment group (20 µg/mouse) was observed (*P* < 0.05). Complete tumor regression in all mice even at the final day (day 50) of the study was observed in 53L10 (20 µg/mouse) treatment group.

Tumor regression by treatment with 53L10 was reproduced also in a different set of the study (Supplementary Fig. 5). The growth of A-549 subcutaneous tumors was again significantly inhibited by 53L10 administration in a dose dependent manner and the complete tumor regression in all mice even at the final day (day 42) was observed in 53L10 (20 µg/mouse) treatment group.

**Fig. 12:**
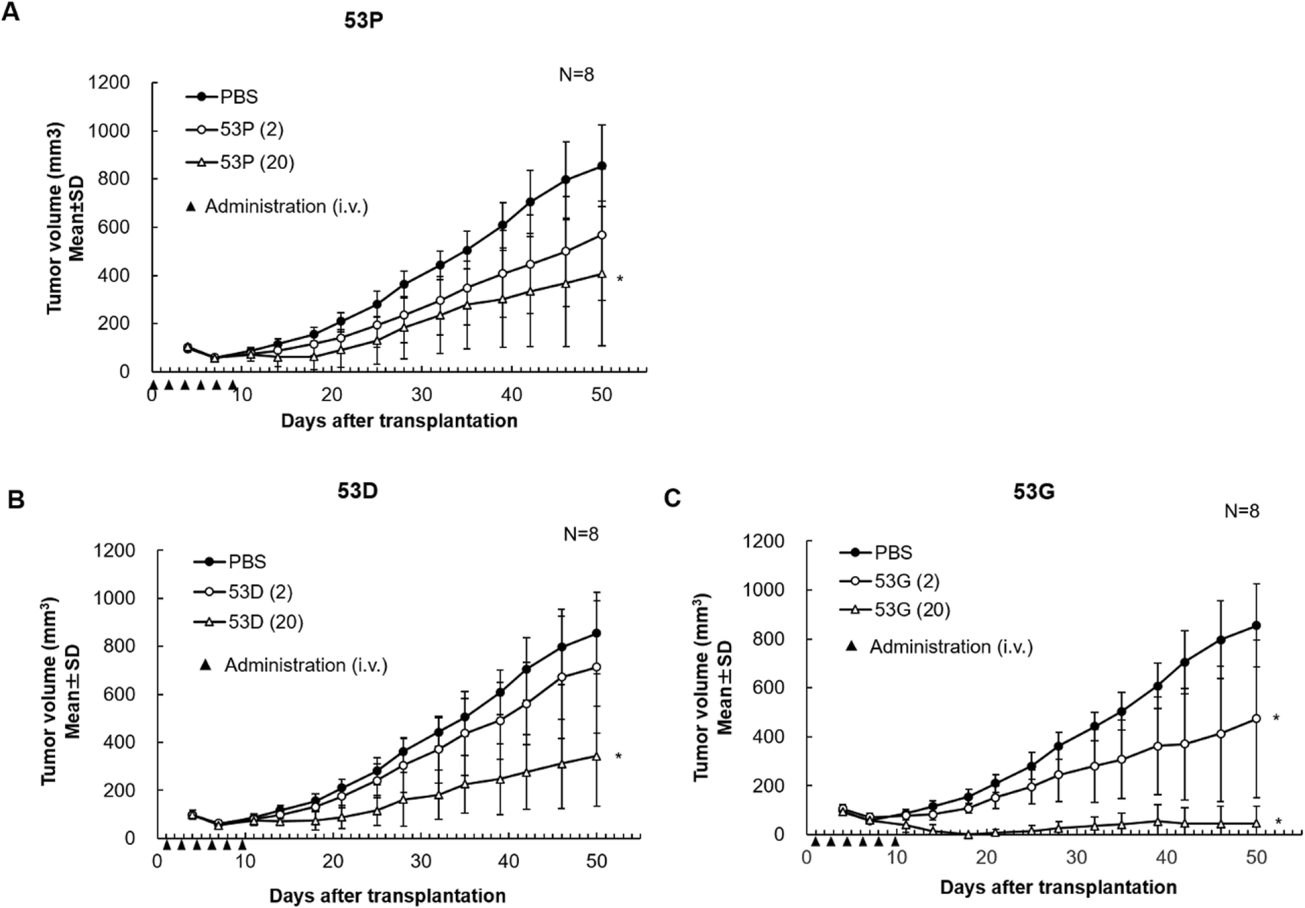
In vivo anti-tumor efficacy of 53D and 53G compared to 53P. Tumor growth curves of A-549 subcutaneous tumors treated in the presence of human PBMCs with (**A**) 53P (negative control), (**B**) 53D, and (**C**) 53G at two different doses (〇: 2 μg/mouse treatment, △: 20 μg/mouse treatment). ●: vehicle (PBS) treatment. Tumor volume was expressed as mean ± standard deviation (SD). *P < 0.05 by Dunnett test (vs vehicle treatment group)

Anti-tumor activities of 53D and 53G, respectively, was also evaluated in the same models in comparison with the control 53P tribody (Fig. 12). Although the patterns of tumor growth inhibition by 53D was similar to that of the control 53P, tumor growth inhibition obtained with 53G treatment (both at 2 µg/ and 20 µg/mouse dose) was significantly stronger compared with vehicle treatment. Significant tumor regression during the study was observed in 53G (20 µg/mouse) treatment group.

## Discussion

Currently, over 70 antibodies have been approved by the FDA, and about 30 of them are in clinical use for the treatment of cancer patients [1–3]. The standard cancer immunotherapy include mono-specific human antibodies with the two binding sites directed against the same target, but a growing interest in cancer immunotherapy is focusing on immune cell engaging bi-specific antibodies (BsAbs), that are antibody-derivative constructs in which the binding sites are directed to different targets, frequently represented by a TAA for one arm and an immune cell receptor for the other one [8]. Among them, Catumaxomab, a BsAbs targeting Epithelial cell adhesion molecule (EpCAM) and CD3, was approved by the European Medicines Agency (EMA) in 2009 for the treatment of malignant ascites [4–6] but the presence of rat/mouse hybrid Fc resulted in significant toxicity. At present, 57 BsAbs are in clinical trials for treatment of cancer patients and, in particular, 38 of them exploit the use of T cell-engaging BsAbs [7, 8].

To avoid the non-specific cytokine storms, recombinant bi-specific derivatives were created lacking the Fc, such as Blinatumomab, which is a BiTE made up of two scFvs targeting CD19 and CD3, that was approved by the FDA in December 2014 and by the EMA in December 2015 for the treatment of the ALL [6, 7]. Despite the success of Blinatumomab against B-cell malignancies expressing CD19, continuous infusion is needed due to the low molecular weight (50 kDa) and a significant portion of patients does not respond to treatment or they eventually relapse even with initial responses. The resistance to Blinatumomab appears to involve multiple mechanisms including immunosuppressive factors that could hamper the effects of immune cell-engaging BsAbs. Indeed, it has been reported that a BsAb, named RO6958688, targeting CEA and CD3, increase T cell infiltration into a xenograft colon carcinoma in mice co-grafted with PBMCs, converting a PD-L1 negative tumor in a PD-L1 positive one [3, 7, 8]. Early results showed enhanced activity of CEA/CD3 targeting BsAb, when combined with the anti-PD-L1 antibody Atezolizumab in patients with metastatic colorectal cancer [33–35]. Therefore, the immunosuppressive signals in TME represent a critical obstacle for effective immunotherapies, suggesting that inhibition of immune checkpoints could potentiate the efficacy of T cell engaging therapy [10, 11, 36].

Hence, we designed and constructed novel tri-specific antibody constructs by combining the specificity of a previous validated bi-specific T cell engager tribody with antibodies targeting different immune checkpoints, in order to obtain in a single molecule, with a larger molecular weight (100 kDa) than BiTEs, the ability to redirect the cytotoxic T cells against cancer cells and that responsible to potentiate the anti-tumor efficacy, due to the immune checkpoint inhibition.

We firstly investigated on novel combinations of multiple immunomodulatory mAbs, recently isolated in our laboratory, specific for PD-L1, PD-1 or LAG-3, able to strong activate T cells [24–28], with the parental bi-specific tribody (Tb535H) targeting the 5T4-TAA on tumor cells and CD3 on T cells, which already demonstrated anti-tumor efficacy in preclinical studies [23].

The promising results of the combined treatments prompted us to generate five novel tri-specific tribodies composed of the 5T4 binding Fab arm, the CD3 binding scFv and an additional scFv derived from an antibody targeting a specific IC. In particular the 53L1 and 53L10 tribodies contain the scFv specific for PD-L1; the 53D and 53G those specific for PD-1 and for LAG-3, respectively, and the 53P, used as negative control, contains an unrelated scFv derived from Palivizumab [31].

The novel tri-specific tribodies were expressed, purified and tested for their binding to human activated T cells, expressing CD3 in its native conformation, to 5T4-expressing tumor cells, and to immobilized recombinant ICs and 5T4. All the novel tribodies were found able to bind to hPBMCs and 5T4-positive tumor cells in a comparable manner or even better than the parental Tb535H, and to retain the binding ability of both the parental mAbs for ICs as well as that of Tb535H for 5T4.

Competitive ELISA assays were then performed to test the ability of the novel tribodies to interfere in the PD-1/PD-L1 or LAG-3/MHCII interactions in comparison with the parental immunomodulatory mAbs. All the novel tribodies confirmed their antagonistic properties in a similar fashion to those of the parental mAbs.

We further tested the novel compounds in cytotoxic assays on co-cultures of breast and lung tumor cells with hPBMCs. The tribodies containing the anti-PD-1 or anti-LAG-3 scFv (53D and 53G) showed up to the 60–70% of tumor cells lysis and those with the anti-PD-L1 scFv (53L1 and 53L10) led to 80–90% of tumor cell lysis. These anti-tumor effects were indeed found to be much more potent than those observed either with parental compounds used alone or in combinations (average tumor lysis of 15–20% and 30–40%, respectively).

As a control for off-target cytotoxic effects on circulating naïve lymphocytes, unstimulated hPBMCs were cultured in the absence of tumor cells and treated with the novel tribodies used at increasing concentrations and for longer time of incubation. The secretion of IL-2 and IFNγ were measured and found to be very low when the tribodies were used in the absence of tumor cells, thus suggesting that the tribodies do not activate naïve T cells not expressing high levels of ICs and do not elicit off target cytokine release.

On the basis of the in vitro promising results, the novel tribodies were tested in vivo at two different doses (2 or 20 µg/mouse) on mice transplanted with A-549 lung tumors together with hPBMCs and found to efficiently inhibit tumor growth with higher efficacy than the parental tribody. More excitingly, the tribodies 53L1 and 53L10, containing the scFv specific for PD-L1 were found able to induce a total regression of tumors not observed with the parental tribody lacking the IC inhibitor.

These findings indicate that the novel 53X tribodies can form immunological synapses between 5T4 on the tumor cells and TCR/CD3 complex on the cytotoxic T cells to activate T cells without HLA/MHC restriction. This could be the major advantage of 53X tribodies compared with soluble TCR therapy for TCR therapy resistant solid tumors. In addition to 5T4-mediated TCR/CD3 activation, 53L10 can also simultaneously inhibit PD-1/PD-L1 interaction, which is an additional advantage for tri-specific tribodies compared with the recently approved bi-specific Tebetafusp [37].

Overall, the data presented in this work strongly indicate that some of the novel tribodies have the potential to become strong and safe therapeutic drugs, due to the addition of immune checkpoints inhibitors that potentiate the cytotoxic effects of T cells against cancer cells. Indeed, they could have some additional advantages with respect to BiTEs, such as the ability to bind to three different targets allowing to reduce also the cost of production as one single molecule contains three different specificities including the anti-TAA, anti-CD3 and anti-IC binding arms, and the higher molecular weight that could allow for longer half-life in circulation as previously reported for other Tribodies [38]. Furthermore, this novel approach could be extended to other T cell engager constructs targeting different TAAs in order to potentiate their cytotoxic effects against cancer cells.

## Conclusions

We generated for the first time novel tri-specific and multi-functional antibody constructs capable of strongly activating T cells against cancer cells and endowed with strong anti-tumor efficacy. In particular, a tri-specific T cell engager, targeting 5T4, CD3 and PD-L1, showed the most promising anti-tumor efficacy in vitro and led to complete tumor regression in vivo.

## Supplementary Information

**Additional file 1: Supplementary Fig. 1.** Cytotoxic effects of the combinations of Tb535H with the parental mAbs on MDA-MB-231 (A), BT-549 (B) or MCF-7 (C) cells co-cultured with hPBMCs. Tumor cells were co-cultured with hPBMCs (Effector:Target cells ratio 5:1) and treated for 48 hours with Tb535H (light grey bars), PD-1_1, LAG-3_1, PD-L1_1, 10_12 immunomodulatory mAbs (dark grey bars) or their combinations (black bars) at the indicated concentrations. Co-cultures untreated or treated with the unrelated mAb (empty bars) were used as negative controls. The release of LDH, as marker of cell lysis, and the secretion of IFNγ, as marker of T cell activation, were measured in supernatants of the co-cultures, as described in Methods. Error bars depict means ± SD. *P*-values are: *** *P* ≤ 0,001; ** *P* < 0.01; * *P* < 0.05. **Supplementary Fig. 2.** Binding curves of parental Tb535H and novel 53X tribodies to human activated PBMCs by cell ELISA. hPBMCs were activated with SEB (50ng/ml) for 48 hours and incubated with increasing concentrations of the tribodies (0 – 20 nM) for 90 min. The binding detection was carried out by using an anti-His HRP-conjugated Ab, as described in Methods. Binding values were reported as the mean of determinations obtained in three independent experiments. Error bars depict means ± SD. **Supplementary Fig. 3.** Binding assays to test the 53G tribody on LAG-3-positive HuT78 cells by cell ELISA. HuT 78 cells were incubated with 53G or the parental LAG-3_1 mAb used at increasing concentrations (10-200 nM). The binding detection was carried out by using an appropriate HRP-conjugated Ab, as described in Methods. Binding values were reported as the mean of determinations obtained in three independent experiments. Error bars depict means ± SD. **Supplementary Fig. 4.** Effects of novel tribodies on lymphocytes in the absence of tumor cells. hPBMCs were treated with the novel tribodies or the parental TB535H at the concentration of 100 pM (grey bars) or 1 nM (black bars) for 66 hours. Lymphocytes untreated or treated with the unrelated mAb or the unrelated tribody 53P were used as negative controls. The secretion of IL-2 and IFNγ was measured by ELISA on supernatants by using the cytokine secretion kit (R & D systems). Error bars depict means ± SD. **Supplementary Fig. 5.** Tumor regression activity of 53L10 tribodies in a different set of the study. Tumor regression activity of 53L10 tribody in A-549 xenograft model. Preparation of A-549 cells and hPBMCs, and subcutaneous transplantation of mixed cells of A-549 and hPBMCs were perfomed as previously described in figure 10. The novel tribody 53L10 at the dosage of 2 μg/mouse (〇) or 20 μg/mouse (△) was administered intravenously on day 0, 2, 4, 6, 8 and 10. Tumor growth in control (no treatment) group (●) was measured in parallel. Tumor volumes were expressed as mean ± standard deviation (SD). *P < 0.05 by Dunnett test (vs vehicle treatment group).

## Data Availability

All data generated or analyzed during this study are included in this manuscript.

## Abbreviations

TCE: T Cell Engagers
TAA: Tumor Associated Antigen
HLA: Human Leucocyte Antigen
BiTE: Bi-specific T Cell Engager
ALL: Acute Lymphoblastic Leukemia
TME: Tumor Microenvironment
MDSCs: Myeloid Derived Suppressor Cells
TAMs: Tumor-Associated Macrophages
Tregs: Regulatory T cells
ICIs: Immune Checkpoint Inhibitors
ATCC: American Type Culture Collection
SEC: Size-Exclusion Chromatography
BSA: Bovine Serum Albumin
hPBMCs: Human Peripheral Blood Mononuclear Cells
SD: Standard Deviation
LDH: Lactate Dehydrogenase
BsAbs: Bi-specific Antibodies
EpCAM: Epithelial Cell Adhesion Molecule
EMA: European Medicines Agency
