# Supplementary material for: Novel tri-specific tribodies induce strong T cell activation and anti-tumor effects in vitro and in vivo
Source: J Exp Clin Cancer Res. 2022 Sep 7;41:269. doi: 10.1186/s13046-022-02474-3 (PMC9450414; doi:10.1186/s13046-022-02474-3)
Supplement: Supplementary file 1 — Additional file 1: Supplementary Fig. 1. Cytotoxic effects of the combinations of Tb535H with the parental mAbs on MDA-MB-231 (A), BT-549 (B) or MCF-7 (C) cells co-cultured with hPBMCs. Tumor cells were co-cultured with hPBMCs (Effector:Target cells ratio 5:1) and treated for 48 hours with Tb535H (light grey bars), PD-1_1, LAG-3_1, PD-L1_1, 10_12 immunomodulatory mAbs (dark grey bars) or their combinations (black bars) at the indicated concentrations. Co-cultures untreated or treated with the unrelated mAb (empty bars) were used as negative controls. The release of LDH, as marker of cell lysis, and the secretion of IFNγ, as marker of T cell activation, were measured in supernatants of the co-cultures, as described in Methods. Error bars depict means ± SD. P-values are: *** P ≤ 0,001; ** P < 0.01; * P < 0.05. Supplementary Fig. 2. Binding curves of parental Tb535H and novel 53X tribodies to human activated PBMCs by cell ELISA. hPBMCs were activated with SEB (50ng/ml) for 48 hours and incubated with increasing concentrations of the tribodies (0 – 20 nM) for 90 min. The binding detection was carried out by using an anti-His HRP-conjugated Ab, as described in Methods. Binding values were reported as the mean of determinations obtained in three independent experiments. Error bars depict means ± SD. Supplementary Fig. 3. Binding assays to test the 53G tribody on LAG-3-positive HuT78 cells by cell ELISA. HuT 78 cells were incubated with 53G or the parental LAG-3_1 mAb used at increasing concentrations (10-200 nM). The binding detection was carried out by using an appropriate HRP-conjugated Ab, as described in Methods. Binding values were reported as the mean of determinations obtained in three independent experiments. Error bars depict means ± SD. Supplementary Fig. 4. Effects of novel tribodies on lymphocytes in the absence of tumor cells. hPBMCs were treated with the novel tribodies or the parental TB535H at the concentration of 100 pM (grey bars) or 1 nM (black bars) fo [file 13046_2022_2474_MOESM1_ESM.docx]

**Supplementary Material**

**Title**: [**Novel Tri-Specific Tribodies induce strong T Cell Activation and anti-Tumor effects**](https://www.scopus.com/record/display.uri?eid=2-s2.0-85126877007&origin=resultslist&sort=plf-f) ***in vitro* and *in vivo***

**Authors**: Margherita Passariello^1,2±^, Asami Yoshioka^3^^±^, Kota Takahashi^3^, Shu-ichi Hashimoto^3^, Toshikazu Inoue^3^, Koji Nakamura^3*^ and Claudia De Lorenzo^1,2,^^*^

^±^These authors contributed equally to this work

**Affiliations**:

1 Department of Molecular Medicine and Medical Biotechnologies, University of Naples “Federico II”, 80131 Naples, Italy

2 Ceinge - Biotecnologie Avanzate s.c. a.r.l., via Gaetano Salvatore 486, 80145 Naples, Italy

3 Chiome Bioscience Inc, 3-12-1 Honmachi Shibuya-Ku, Tokyo 151-0071 Japan

*Correspondence: cladelor@unina.it; Phone: +39-081-3737868; knakamura@chiome.co.jp; Phone: +81-44-766-2119

**Supplementary Figures and Legends**

**
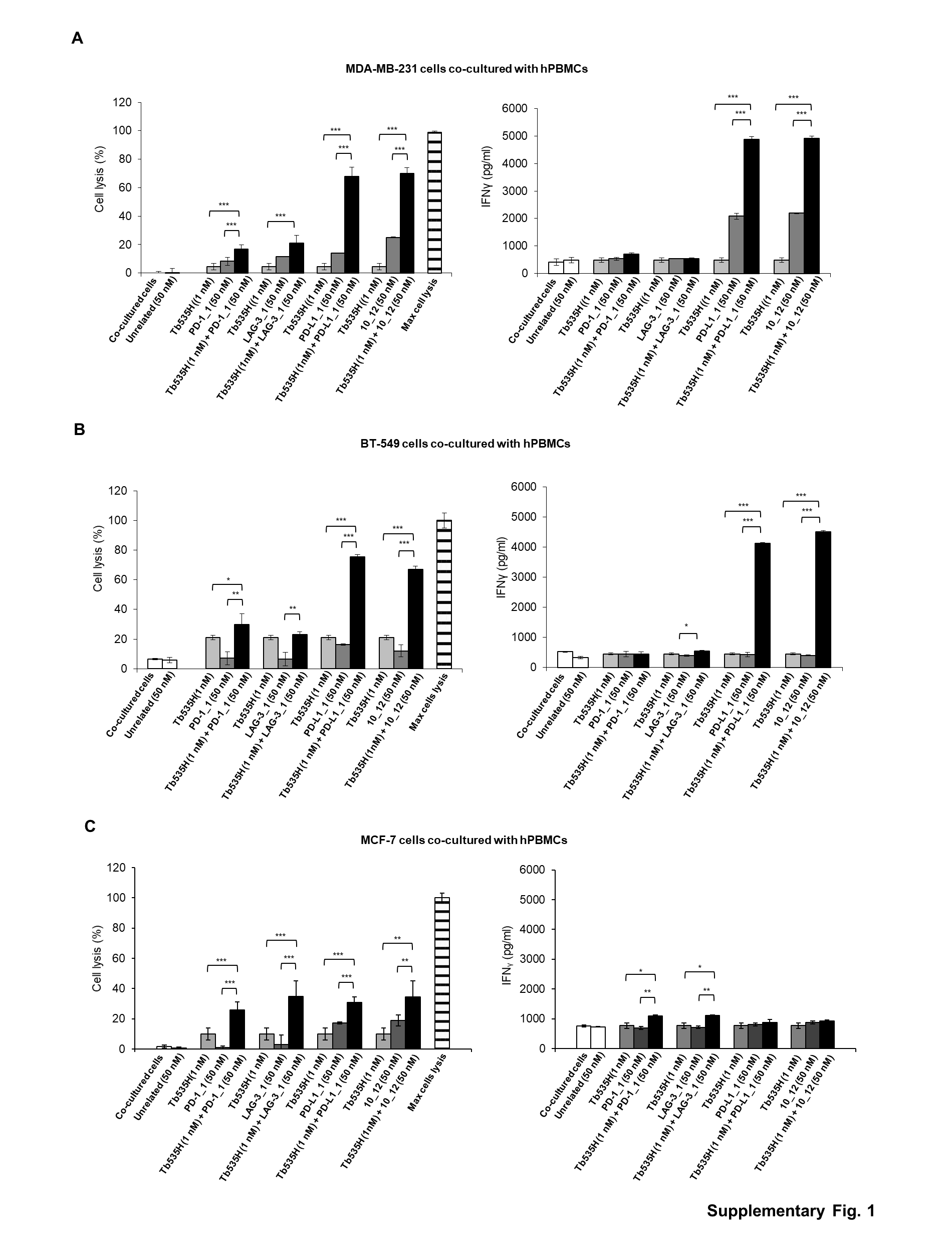
**

**Supplementary Fig. 1.** Cytotoxic effects of the combinations of Tb535H with the parental mAbs on MDA-MB-231 (**A**), BT-549 (**B**) or MCF-7 (**C**) cells co-cultured with hPBMCs. Tumor cells were co-cultured with hPBMCs (Effector:Target cells ratio 5:1) and treated for 48 hours with Tb535H (light grey bars), PD-1_1, LAG-3_1, PD-L1_1, 10_12 immunomodulatory mAbs (dark grey bars) or their combinations (black bars) at the indicated concentrations. Co-cultures untreated or treated with the unrelated mAb (empty bars) were used as negative controls. The release of LDH, as marker of cell lysis, and the secretion of IFNγ, as marker of T cell activation, were measured in supernatants of the co-cultures, as described in Methods. Error bars depict means ± SD. P-values are: *** P ≤ 0,001; ** P < 0.01; * P < 0.05.

**
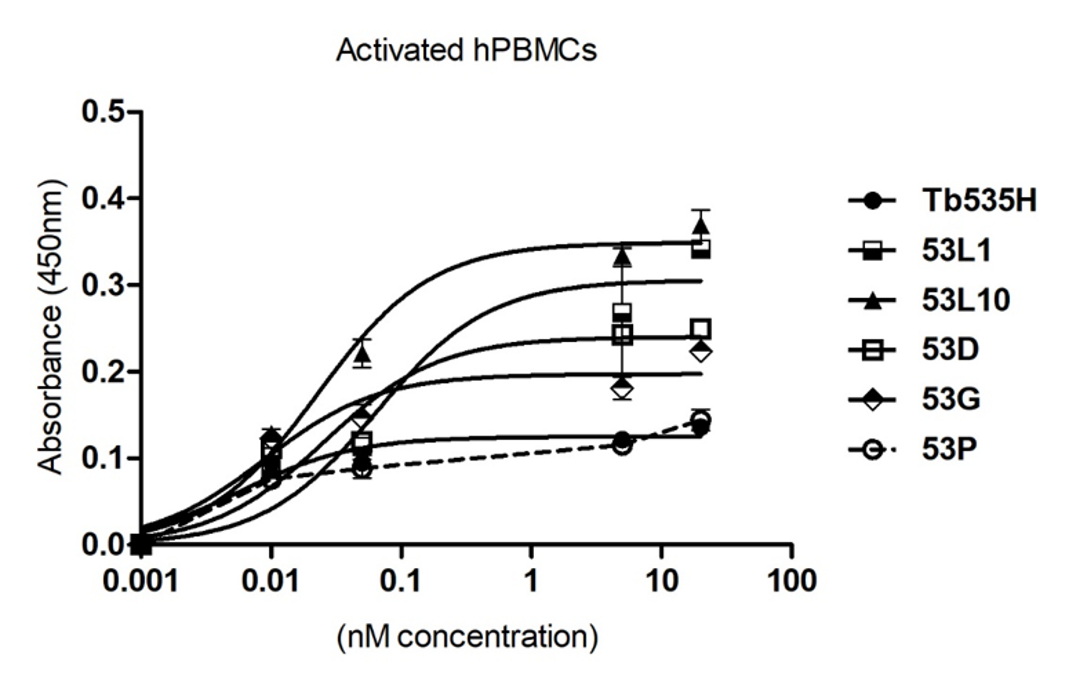
**

**Supplementary Fig. 2.** Binding curves of parental Tb535H and novel 53X tribodies to human activated PBMCs by cell ELISA. hPBMCs were activated with SEB (50ng/ml) for 48 hours and incubated with increasing concentrations of the tribodies (0 – 20 nM) for 90 min. The binding detection was carried out by using an anti-His HRP-conjugated Ab, as described in Methods. Binding values were reported as the mean of determinations obtained in three independent experiments. Error bars depict means ± SD.

**
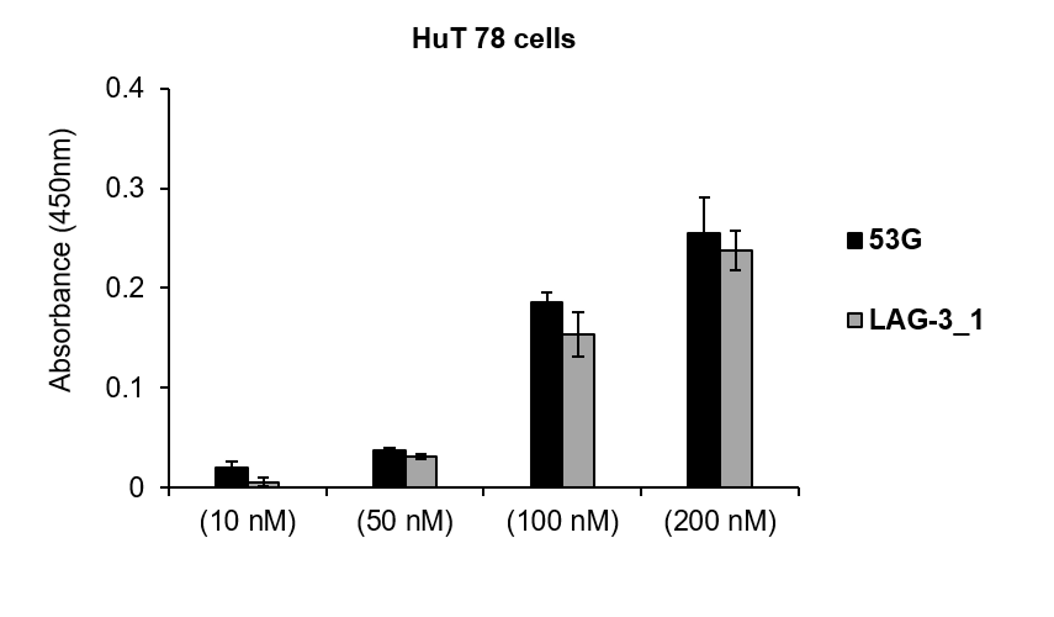
**

**Supplementary Fig. 3.** Binding assays to test the 53G tribody on LAG-3-positive HuT78 cells by cell ELISA. HuT 78 cells were incubated with 53G or the parental LAG-3_1 mAb used at increasing concentrations (10-200 nM). The binding detection was carried out by using an appropriate HRP-conjugated Ab, as described in Methods. Binding values were reported as the mean of determinations obtained in three independent experiments. Error bars depict means ± SD.


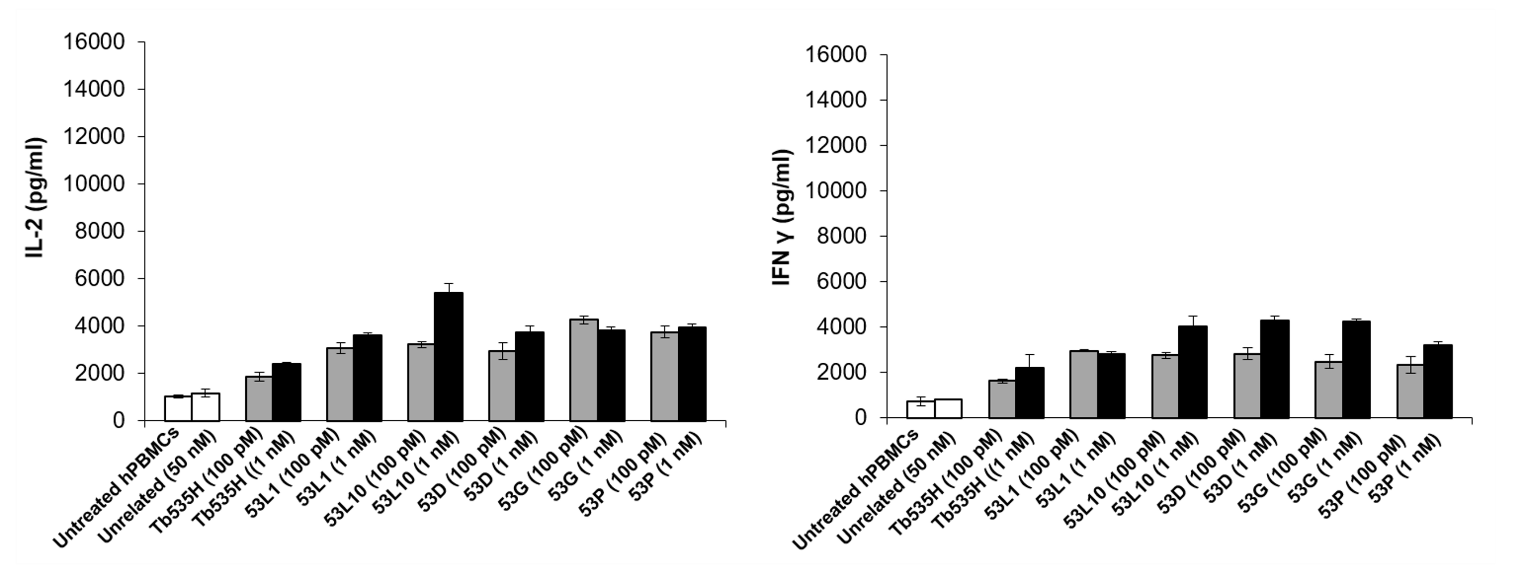


**Supplementary Fig. 4.** Effects of novel tribodies on lymphocytes in the absence of tumor cells. hPBMCs were treated with the novel tribodies or the parental TB535H at the concentration of 100 pM (grey bars) or 1 nM (black bars) for 66 hours. Lymphocytes untreated or treated with the unrelated mAb or the unrelated tribody 53P were used as negative controls. The secretion of IL-2 and IFNγ was measured by ELISA on supernatants by using the cytokine secretion kit (R & D systems). Error bars depict means ± SD.

**
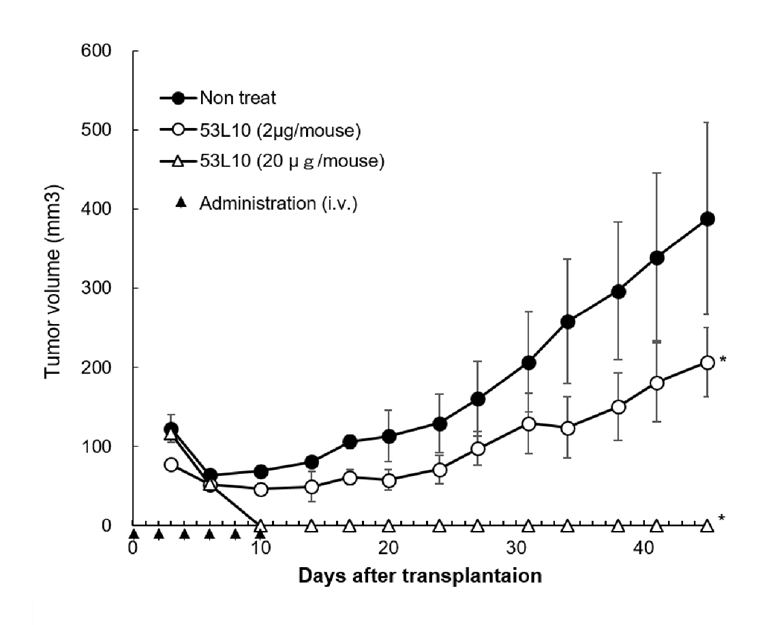
**

**Supplementary Fig. 5. Tumor regression activity of 53L10 tribodies in a different set of the study.** Tumor regression activity of 53L10 tribody in A-549 xenograft model. Preparation of A-549 cells and hPBMCs, and subcutaneous transplantation of mixed cells of A-549 and hPBMCs were perfomed as previously described in figure 10. The novel tribody 53L10 at the dosage of 2 μg/mouse (〇) or 20 μg/mouse (△) was administered intravenously on day 0, 2, 4, 6, 8 and 10. Tumor growth in control (no treatment) group (●) was measured in parallel. Tumor volumes were expressed as mean ± standard deviation (SD). *P < 0.05 by Dunnett test (vs vehicle treatment group).
